# The Oncogenic Transcription Factor RUNX1/ETO Corrupts Cell Cycle Regulation to Drive Leukemic Transformation

**DOI:** 10.1016/j.ccell.2018.08.015

**Published:** 2018-10-08

**Authors:** Natalia Martinez-Soria, Lynsey McKenzie, Julia Draper, Anetta Ptasinska, Hasan Issa, Sandeep Potluri, Helen J. Blair, Anna Pickin, Asmida Isa, Paulynn Suyin Chin, Ricky Tirtakusuma, Daniel Coleman, Sirintra Nakjang, Salam Assi, Victoria Forster, Mojgan Reza, Ed Law, Philip Berry, Dorothee Mueller, Alex Elder, Simon N. Bomken, Deepali Pal, James M. Allan, Gareth J. Veal, Peter N. Cockerill, Christian Wichmann, Josef Vormoor, Georges Lacaud, Constanze Bonifer, Olaf Heidenreich

**Affiliations:** 1 Wolfson Childhood Cancer Research Centre, Northern Institute for Cancer Research, Newcastle University, Brewery Lane, Newcastle upon Tyne NE1 7RU, UK; 2 Cancer Research UK Manchester Institute, Manchester M20 4GJ, UK; 3 Institute for Cancer and Genomic Sciences, College of Medical and Dental Sciences, University of Birmingham, Birmingham B15 2TT, UK; 4 Newcastle Cancer Centre, Northern Institute for Cancer Research, Newcastle University, Newcastle upon Tyne NE2 4HH, UK; 5 Department of Transfusion Medicine, Cell Therapeutics and Hemostaseology, Ludwig-Maximilian University Hospital, Munich 80539, Germany; 6 Princess Maxima Center for Pediatric Oncology, Utrecht 3584CS, the Netherlands

**Keywords:** RNAi screen, fusion gene, cell-cycle control, CDK6 inhibition, RUNX1/ETO, CCND2, acute myeloid leukemia, palbociclib, KIT mutation, imatinib

## Abstract

Oncogenic transcription factors such as the leukemic fusion protein RUNX1/ETO, which drives t(8;21) acute myeloid leukemia (AML), constitute cancer-specific but highly challenging therapeutic targets. We used epigenomic profiling data for an RNAi screen to interrogate the transcriptional network maintaining t(8;21) AML. This strategy identified Cyclin D2 (CCND2) as a crucial transmitter of RUNX1/ETO-driven leukemic propagation. RUNX1/ETO cooperates with AP-1 to drive *CCND2* expression. Knockdown or pharmacological inhibition of CCND2 by an approved drug significantly impairs leukemic expansion of patient-derived AML cells and engraftment in immunodeficient murine hosts. Our data demonstrate that RUNX1/ETO maintains leukemia by promoting cell cycle progression and identifies G1 CCND-CDK complexes as promising therapeutic targets for treatment of RUNX1/ETO-driven AML.

## Highlights

• An RNAi screen identifies *CCND2* as a crucial transcriptional target of RUNX1/ETO
• RUNX1/ETO promotes *CCND2* expression by binding to an upstream element
• CCND2 knockdown inhibits RUNX1/ETO-driven leukemic expansion *in vitro* and *in vivo*
• RUNX1/ETO-expressing leukemic cells are highly sensitive to a CDK4/6 inhibitor

## Graphical Abstract

**Figure:**
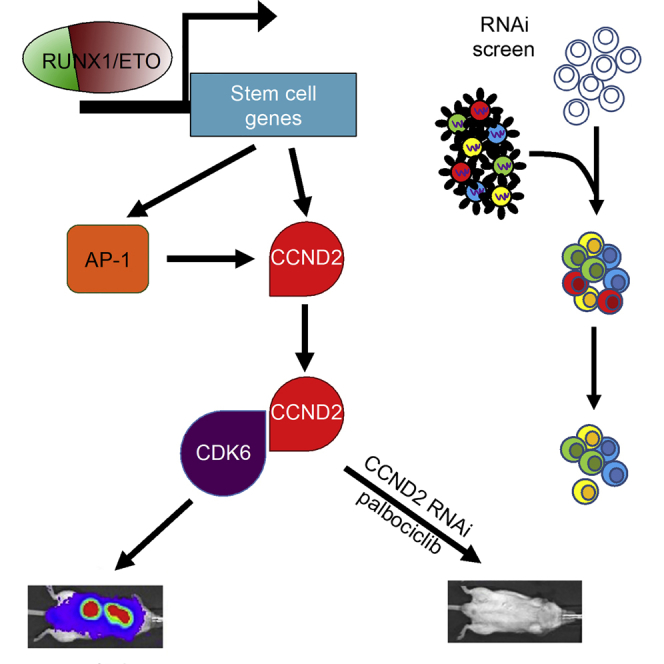

## Teaser

Using *in vitro* and *in vivo* screens to identify essential RUNX1/ETO transcriptional targets in AML, Martinez-Soria identify *CCND2* as required for leukemia maintenance and self-renewal. Targeting this dependency using the CDK4/6 inhibitor palbociclib prolongs the survival of AML PDX models.

## Significance

**Leukemic fusion proteins drive leukemia by maintaining abnormal transcriptional networks. In contrast to most fusion proteins themselves, network components relaying fusion protein function may be amenable to pharmacologic interference. We tested this hypothesis by using an RNAi screen to functionally interrogate transcriptional targets of the fusion protein RUNX1/ETO for their relevance for leukemia maintenance. This approach identified the cell-cycle regulator *CCND2* as an essential RUNX1/ETO target gene, which confers high sensitivity toward palbociclib, a clinically approved inhibitor of CCND-CDK4/6 complexes. This study demonstrates the feasibility of epigenomics-instructed screens for identifying oncogene-driven vulnerabilities and their exploitation by repurposed drug approaches.**

## Introduction

Therapeutic exploitation of oncogene addiction has become a central aim of modern cancer therapy, but effective targeted therapies have yet to be developed for the majority of acute leukemia subtypes. Many of these are caused by chromosomal rearrangements generating aberrant transcriptional regulators such as RUNX1/ETO (Miyoshi et al., 1993). Treatments generally involve intensive and genotoxic chemotherapy, which can severely impair the quality of life of patients during treatment and of long-term survivors (de Rooij et al., 2015). The toxicity of current treatments and the dissatisfactory long-term survival of less than 70% even in acute myeloid leukemia (AML) subgroups with “good prognosis” demand therapeutic concepts for more precise interference with the leukemic program.

The chromosomal translocation t(8;21) generates the RUNX1/ETO fusion protein, which interferes with normal hematopoiesis by deregulating the expression of hundreds of genes, many of them bound by the fusion protein and its binding partners, thus defining a core transcriptional network of RUNX1/ETO-responsive genes (Martens et al., 2012, Ptasinska et al., 2012, Ptasinska et al., 2014). We reasoned that such a transcriptional network contains crucial mediators of a fusion protein-driven AML maintenance program that are amenable to pharmacological inhibition. Therefore, we tested the idea that RUNX/1ETO generates addictions for malignant cells accessible to therapeutic intervention.

## Results

### An RNAi Screen Identifies RUNX1/ETO Target Genes Essential for Leukemic Propagation

**Figure 1:**
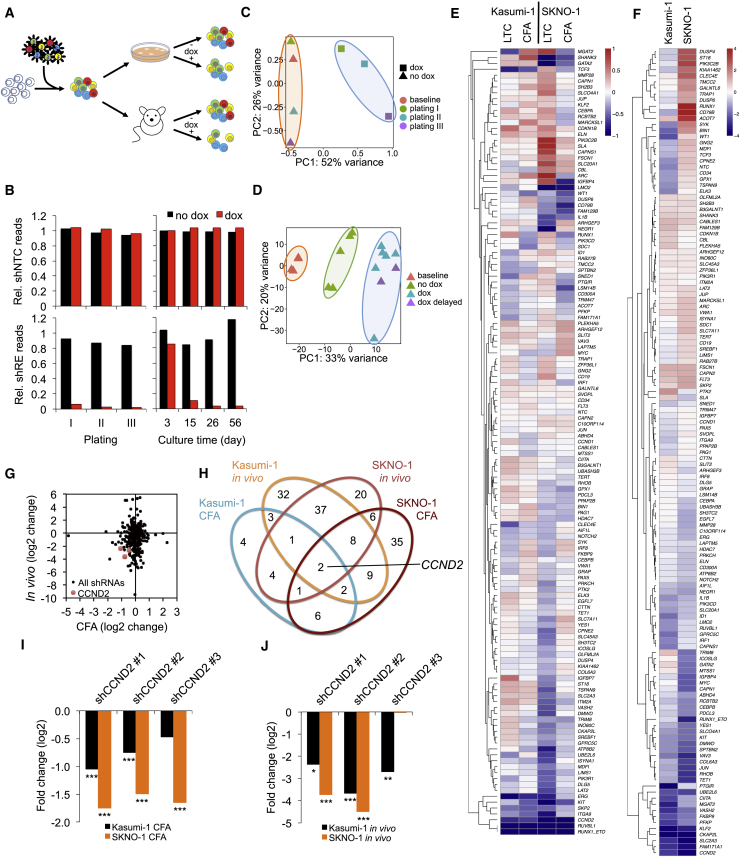
A Combined *In Vitro*/*In Vivo* RNAi Screen Identifies *CCND2* as Crucial Mediator of RUNX1/ETO Function (A) Scheme of the RNAi screen. t(8;21) cell lines were transduced with the lentiviral shRNA library and propagated with and without shRNA induction by doxycycline either *in vitro* in three consecutive replatings (12–14 days per plating) and long-term suspension culture for up to 56 days (LTC) or *in vivo* by xenotransplantation of immunodeficient mice killed upon reaching clinical endpoints. (B) Changes in relative (Rel.) sequencing read levels of proviral non-targeting control shRNA (shNTC) and RUNX1/ETO shRNA (shRE). (C) PCA of shRNA pools in Kasumi-1 colony formation assay (CFA) cells during replating. PC, principal component. (D) PCA of shRNA pools from Kasumi-1 transplanted NSG mice. dox, dox treatment initiated immediately after transplantation; dox delayed, doxycycline treatment initiated 28 days after transplantation. (E and F) Clustered heatmaps showing fold changes for genes in the *in vitro* (E) and the *in vivo* (F) arms of the RNAi screen. Fold changes were calculated based on collapsed changes of shRNAs using the RRA approach of MAGeCK. (G) Comparison of changes in shRNA construct levels *in vivo* and after the third replating. (H) Venn diagram identifying depleted shRNA constructs shared between the different RNAi screen conditions. (I and J) Fold change of all *CCND2* shRNA constructs after third replatings (I) and *in vivo* engraftment (J). ^∗∗∗^p < 0.001; ^∗∗^p < 0.01; ^∗^p < 0.05 compared with no dox controls. See also Figure S1 and Tables S1, S2, and S3.

To identify pathways essential for RUNX1/ETO-driven leukemogenesis, we performed an RNAi screen targeting RUNX1/ETO-bound genes responsive to RUNX1/ETO depletion (Figure 1A) (Ptasinska et al., 2012, Ptasinska et al., 2014). Gene set enrichment analysis (GSEA) linked the set of genes downregulated by RUNX1/ETO depletion to self-renewal programs (Figure S1A) (Ben-Porath et al., 2008, Jaatinen et al., 2006, Muller et al., 2008). Integration of bead array gene expression data from t(8;21) cell lines and patient material with chromatin immunoprecipitation (ChIP) sequencing (ChIP-seq) data from our perturbation studies defined a set of 110 gene loci bound by RUNX1/ETO and with reduced expression upon RUNX1/ETO knockdown (Ptasinska et al., 2012). Inclusion of negative and positive control constructs and small hairpin RNAs (shRNAs) against genes known to cooperate with RUNX1/ETO, such as *KIT*, *RUVBL1* (also known as Pontin), and *CAPN1*, yielded a lentiviral library of 374 shRNA constructs targeting 133 genes (Table S1) (Breig et al., 2014, Osman et al., 2009, Wichmann et al., 2015). To exclude cell type bias, all screens were performed with two t(8;21) AML cell lines, Kasumi-1 and SKNO-1. Furthermore, the RNAi screens consisted of non-induced and doxycycline-induced arms with 4 × 10^6^ transduced cells in each arm yielding a 10,000-fold coverage of the shRNA library. Differentially expressed shRNA constructs were identified by comparison between the two corresponding arms (Figure 1A). Doxycycline treatment resulted in robust shRNA-associated red fluorescent protein (RFP) expression *in vitro* and *in vivo* (Figures S1B and S1C).

To identify genes required for leukemic self-renewal *in vitro*, we determined changes in shRNA pool compositions after extended suspension culture for up to 56 days or in colony formation assays after three replatings. For the *in vivo* screen, we intrafemorally transplanted NOD.Cg-Prkdc^scid^ Il2rg^tm1Wjl^/SzJ (NSG) mice with either Kasumi-1 or SKNO-1 cells transduced with the RNAi library. Next-generation sequencing yielded 4 × 10^4^ to 2 × 10^6^ reads per pool with 100–5,000 reads per shRNA construct (Figure S1D, Tables S2 and S3). The shRNA construct targeting RUNX1/ETO served as a positive control and was strongly depleted after the first plating. The non-targeting control shNTC remained stable over the course of three replatings in both Kasumi-1 and SKNO-1 cells, thus demonstrating overall functionality of the screen (Figures 1B and S1E).

Principal component analyses (PCAs) demonstrated that doxycycline treatment increased separation of shRNA-expressing cell populations from baseline and untreated samples in all *in vitro* and *in vivo* screens (Figures 1C, 1D, S1F, and S1G). Unlike SKNO-1, engraftment of Kasumi-1 cells in NSG mice induced a modest deviation of the engrafted pool from the baseline pool composition (Figures 1D and S1G). Possible reasons for this shift may include leaky expression of shRNAs in the absence of doxycycline or potential niche competition. Nevertheless, the strategy of pre-selecting potential components of the RUNX1/ETO-driven transcriptional network yielded an extraordinarily high number of hits with more substantial changes in the *in vivo* arm compared with the *in vitro* arm (Figures S1H and S1I). Intersection of depleted shRNA sequences in both replating and *in vivo* screens showed that Kasumi-1 cells shared more than 40% of depleted shRNAs with SKNO-1 cells, indicating a substantial qualitative concordance between t(8;21) cell lines (Figure S1J).

Notably, the *in vitro* and the *in vivo* screens identified distinct groups of genes relevant for RUNX1/ETO-driven leukemia propagation, suggesting that the *in vivo* environment required additional gene functions for successful engraftment (Figures 1E and 1F). Two shRNAs were significantly depleted in all *in vitro* and *in vivo* screens, both of which target *CCND2* (Figures 1G and 1H). All three *CCND2* shRNAs present in the pool were depleted in both cell lines during replating and long-term culture (Figure 1I). Furthermore, all three shRNAs were depleted in Kasumi-1 cells *in vivo*, and two were depleted in SKNO-1 cells *in vivo* (Figure 1J). These data highlight the importance of *CCND2* expression for leukemic propagation *in vitro* and *in vivo*.

### RUNX1/ETO Regulates CCND2 Transcription via an Intergenic Element

*CCND2* is highly expressed with similar transcript levels found in t(8;21)-positive and -negative AML patients (Figures S2A and S2B). To ascertain whether *CCND2* is a transcriptional target of RUNX1/ETO, we analyzed the *CCND2* locus by integrating ChIP-seq and DNaseI hypersensitivity site sequencing (DHS-seq) data (Ptasinska et al., 2012, Ptasinska et al., 2014).

**Figure 2:**
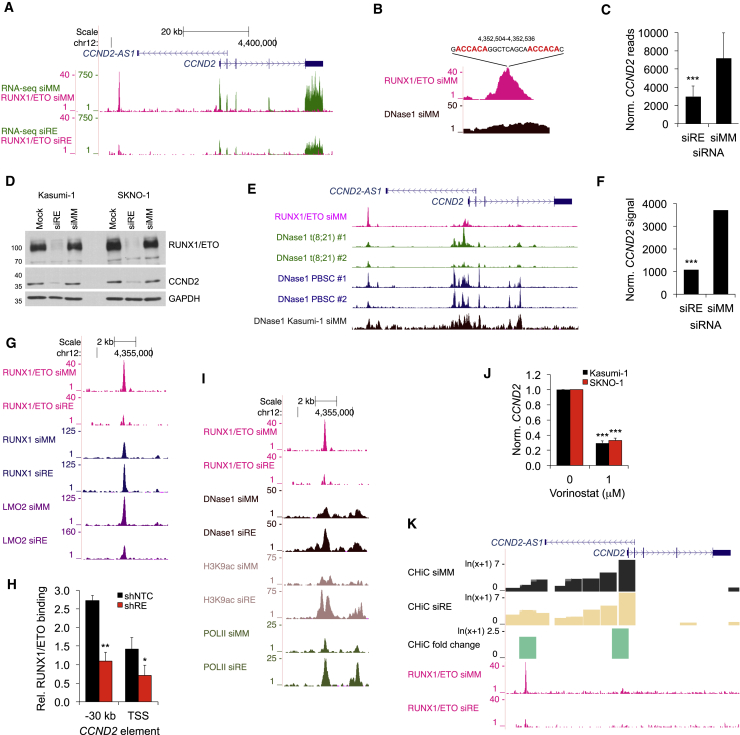
RUNX1/ETO Controls *CCND2* Expression via an Upstream Regulatory Element (A) University of California, Santa Cruz (UCSC) genome browser screenshot displaying changes in transcript levels (green) based on RNA sequencing (RNA-seq) and RUNX1/ETO binding (pink) based on ChIP-seq at the *CCND2* locus in Kasumi-1 cells. siRE, RUNX1/ETO small interfering RNA (siRNA); siMM, mismatch control siRNA. Scale and nucleotide positions are indicated at the top. (B) Screenshot of RUNX1/ETO binding and DNase1 hypersensitive sites (DNase1) at the −30 kb region of *CCND2* in Kasumi-1 cells treated with siMM. The location and sequence are shown on top with the RUNX1 consensus sites indicated in red. (C) Change in *CCND2* transcript levels between Kasumi-1 cells treated with siMM and siRE, as determined by RNA-seq. ^∗∗∗^p < 0.001 compared with siMM. (D) Immunoblots of CCND2 protein levels in Kasumi-1 and SKNO-1 cells following RUNX1/ETO knockdown. Mock, electroporated without siRNA. (E) Chromatin accessibility at *CCND2* for two t(8;21) AML patients (t(8;21) #1 and #2), normal CD34^+^ PBSCs from two donors (PBSC #1 and #2) and Kasumi-1 cells as judged by DHS-seq. Top panel, RUNX1/ETO binding by ChIP-seq. (F) *CCND2* transcript levels in primary AML (patient sample L852) with (siRE) and without (siMM) RUNX1/ETO knockdown as analyzed by Illumina bead arrays with probe ILMN_2067656. ^∗∗∗^p < 0.001 (Illumina custom false discovery rate [FDR]) compared with siMM. (G) Effect of RUNX1/ETO knockdown on transcription factor binding at the *CCND2* locus in Kasumi-1 cells. (H) Assessment of RUNX1/ETO in control (shNTC) or RUNX1/ETO knockdown (shRE) Kasumi-1 cells at the −30 kb element of *CCND2* by manual ChIP. n = 3; mean ± SD; ^∗^p < 0.05; ^∗∗^p < 0.01 compared with shNTC. (I and J) (I) Assessment of epigenetic changes by ChIP-seq and DHS-seq in chromatin structure, histone K9 acetylation, and RNA Pol II occupation at the −30 kb element upon RUNX1/ETO knockdown (siRE) in comparison with siMM in Kasumi-1 cells. (J) Impact of the HDAC inhibitor vorinostat on *CCND2* RNA expression normalized to *GAPDH* (norm. *CCND2*) in t(8;21) AML cell lines. n = 3; mean ± SD; ^∗∗∗^p < 0.001 compared with shNTC. (K) Genome browser screenshot of promoter capture CHiC in Kasumi-1 cells visualizing the impact of RUNX1/ETO depletion on the interaction of the −30 kb element with the *CCND2* TSS. Control siRNA treatment, CHiC siMM; RUNX1/ETO knockdown, CHiC siRE. CHiC fold change, fold difference in interaction between RUNX1/ETO knockdown and control. See also Figure S2.

In t(8;21) Kasumi-1 cells, DHS-seq, and ChIP-seq highlighted an open chromatin region located 30 kb upstream of the *CCND2* transcriptional start site (TSS) occupied by RUNX1/ETO (Figures 2A and S2C) that contained a tandem arrangement of RUNX1 consensus binding sites known to favor RUNX1/ETO occupation (Figure 2B) (Okumura et al., 2008). Knockdown of RUNX1/ETO eliminated its binding to this element and decreased *CCND2* RNA and protein levels (Figures 2A, 2C, 2D, S2C, and S2D). DHS-seq analysis of primary cells from two t(8;21) AML patients and normal peripheral blood stem cells (PBSCs) from healthy donors showed a highly similar pattern to that in Kasumi-1 cells (Figure 2E). Furthermore, RUNX1/ETO knockdown diminished *CCND2* expression in primary AML blasts (Figure 2F). Therefore, *CCND2* regulation is conserved across t(8;21) cell lines and primary patient cells supporting Kasumi-1 cells as an appropriate model system for investigating RUNX1/ETO-exerted control of *CCND2* expression.

Loss of RUNX1/ETO enhanced RUNX1 binding and diminished LMO2 binding at the −30 kb element, indicating competition between fusion and wild-type protein and a preferred interaction of LMO2 with RUNX1/ETO at this site (Figures 2G, 2H, and S2E). These changes were associated with increased DNaseI accessibility at the −30 kb element and increased H3K9 acetylation at both the −30 kb element and the TSS (Figure 2I). Surprisingly, although a mark for actively transcribed promoters, this increase in H3K9 acetylation was linked to reduced RNA polymerase II (RNA pol II) occupancy across the *CCND2* gene body (Figure S2C). However, we observed increased RNA pol II binding at the −30 kb element and a second location at −27 kb, suggesting that increased histone acetylation and RNA pol II occupation at the two upstream elements impairs *CCND2* expression (Figure 2I). Since RUNX1/ETO recruits class I histone deacetylases (HDACs) to DNA (Gelmetti et al., 1998, Lutterbach et al., 1998), we tested the impact of histone acetylation on *CCND2* expression by pharmacological HDAC inhibition. Treatment of Kasumi-1 or SKNO-1 cells with the HDAC inhibitor vorinostat reduced *CCND2* expression, confirming an inhibitory function of histone acetylation of this gene (Figure 2J). Interestingly, genome-wide chromosome conformation capture data (CHiC) from Kasumi-1 cells indicated an interaction between the −30 kb region and the TSS, which was enhanced by RUNX1/ETO loss (Figure 2K). In conclusion, RUNX1/ETO maintains *CCND2* expression by binding to the −30 kb element, which affects the three-dimensional interaction between this element and the *CCND2* TSS.

### RUNX1/ETO Drives CCND2 Expression through AP-1 Factors

**Figure 3:**
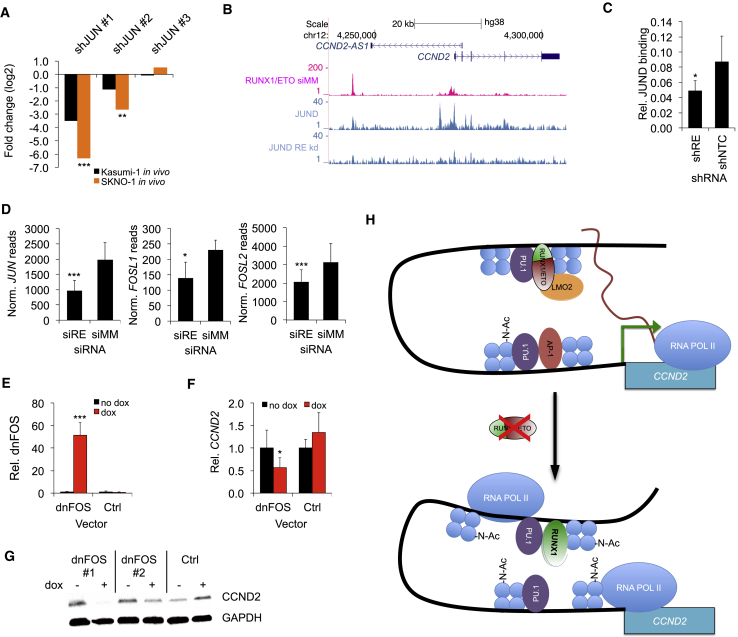
RUNX1/ETO Regulates *CCND2* Expression by Promoting AP-1 Activity (A) Log fold change of three JUN shRNA construct levels in *in vivo* screens in Kasumi-1 and SKNO-1 cells. ^∗∗∗^p < 0.001; ^∗∗^p < 0.01 compared with no dox controls. (B) UCSC screenshot showing JUND and RUNX1/ETO binding to the *CCND2* locus with and without RUNX1/ETO knockdown in Kasumi-1 cells. (C–E) (C) Manual ChIP validation of JUND binding at the *CCND2* promoter with and without RUNX1/ETO knockdown in Kasumi-1 cells. shRE, RUNX1/ETO shRNA; shNTC, non-targeting control shRNA. n = 3; mean ± SD; ^∗^p < 0.05 compared with shNTC. (D) Changes in transcript levels of JUN and FOS members upon RUNX1/ETO knockdown in Kasumi-1 cells as assessed by RNA-seq. Mean ± SD; n = 3. ^∗∗∗^p < 0.001; ^∗^p < 0.05 compared with shNTC. (E) Expression of dnFOS transcript in Kasumi-1 cells lentivirally transduced with dnFOS or control (Ctrl) vector. Cells were incubated for 5 days with and without dox. n = 3; mean ± SD; ^∗∗∗^p < 0.001. (F) Impact of dnFOS induction by doxycycline in Kasumi-1 cells on relative *CCND2* transcript levels measured by qPCR normalized to *GAPDH*. n = 3; mean ± SD; ^∗^p < 0.05. (G) Immunoblot showing CCND2 protein levels in Kasumi-1 cells upon dnFOS induction for 5 days. dnFOS#1 and dnFOS#2, FOS overexpressing clones 1 and 2, respectively; Ctrl, normal Kasumi-1 cells. (H) Scheme depicting a model for the regulation of *CCND2* by RUNX1/ETO. Depletion of RUNX1/ETO enhances interaction between −30 kb and TSS, increases H3K9 acetylation and occupation of the −30 kb element by RUNX1 and RNA Pol II, impairs AP1 binding at the promoter, and stalls RNA Pol II at the TSS, leading to reduced *CCND2* transcription. See also Figure S3.

Next, we asked whether RUNX1/ETO occupancy affected the association of activating transcription factors to the *CCND2* locus, focusing on AP-1, a heterodimer between JUN and FOS family members known to transcriptionally activate *CCND2* (Mathas et al., 2002). *JUN* (c-JUN) is induced upon RUNX1/ETO expression (Figure S3A) (Elsasser et al., 2003), and it scored in the *in vivo* RNAi screen as being essential for leukemia propagation (Figure 3A). Depletion of RUNX1/ETO reduced binding of JUND to the *CCND2* promoter and reduced expression of *JUN* and several *FOS* family members (Figures 3B–3D and S3B). To functionally interfere with all AP-1 heterodimers, we expressed a doxycycline-inducible dominant-negative FOS (dnFOS) (Olive et al., 1997). Induction of dnFOS reduced JUND binding to the *CCND2* promoter and diminished CCND2 transcript and protein levels without interfering with RUNX1/ETO occupation of the −30 kb element (Figures 3E–3G, S3C, and S3D).

These combined data support a model where RUNX1/ETO drives *CCND2* expression by directly binding to the −30 kb element and indirectly by supporting expression and binding of AP-1 family members to the *CCND2* promoter (Figure 3H). Following RUNX1/ETO depletion, RUNX1 binding increases, AP-1 binding is lost, and the balance is shifted to the inactive state of *CCND2*.

### CCND2 Is Required for Propagation of t(8;21)-Positive AML

**Figure 4:**
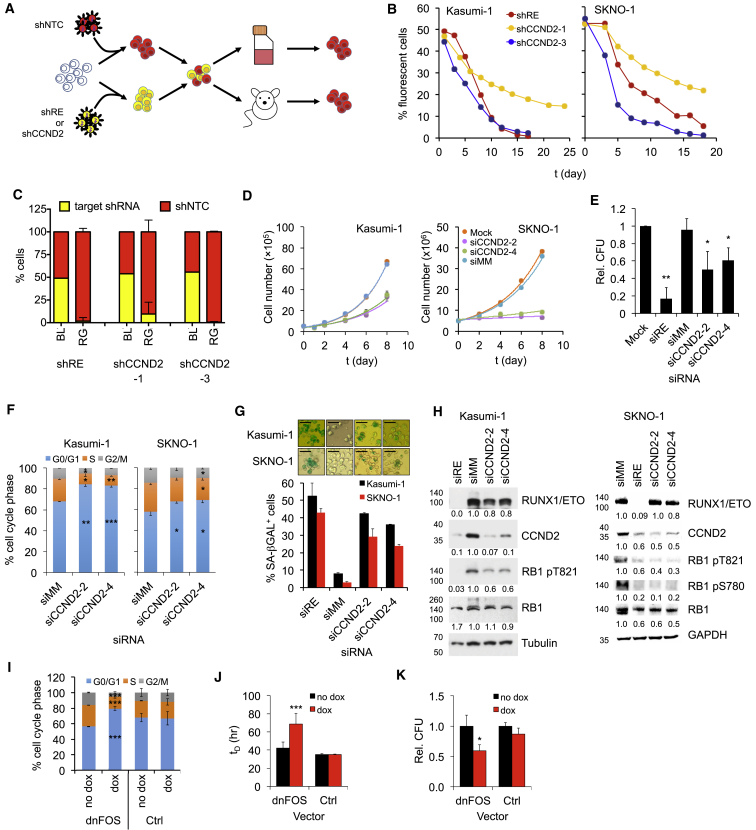
RUNX1/ETO-Expressing AML Cells Are Addicted to CCND2 (A) Scheme of the competitive co-culture and transplantation approaches. t(8;21) cells were lentivirally transduced with either a vector linking RPF657 to a non-targeting control shRNA (shNTC) or dTomato to an shRNA targeting either RUNX1/ETO (shRE) or CCND2 (shCCND2-1, −3). Control and knockdown cells were mixed 50:50 followed by co-culture (Kasumi-1 and SKNO-1) or intrahepatic transplantation into newborn RG mice (Kasumi-1). shRE, RUNX1/ETO shRNA; shCCND2, CCND2 shRNA; shNTC, non-targeting control shRNA. (B) Graph showing percentage of shRE-, shCCND2-1- or shCCND2-3 expressing Kasumi-1 and SKNO-1 cells compared with snNTC expressing cells during LTC. (C) Percentage of Kasumi-1 cells with indicated shRNA in transplanted RG mice. BL, starting pool prior to transplantation; RG, cells harvested from transplanted RG mice humanely killed at clinical endpoints. Mean ± SD, n = 5. (D) Proliferation curves for Kasumi-1 and SKNO-1 cells electroporated sequentially every two days with the indicated siRNAs. Mock; non-siRNA electroporated cells; siCCND2, CCND2 siRNA; siMM, mismatch control siRNA. Kasumi-1, n = 3, mean ± SD; SKNO-1, n = 1. (E) Colony formation of Kasumi-1 cells transduced with the indicated siRNA constructs at 12 days post plating. CFU, colony-forming unit. Mean ± SD; Kasumi-1, n = 3; ^∗∗^p < 0.01; ^∗^p < 0.05 compared with Mock. (F) Cell cycle distribution of Kasumi-1 and SKNO-1 cells with and without CCND2 knockdown. Mean ± SD; n = 3. Counts at 12 days post plating. Mean ± SD; Kasumi-1, n = 3; ^∗∗∗^p < 0.001; ^∗∗^p < 0.01; ^∗^p < 0.05 compared with siMM. (G) Senescence in Kasumi-1 and SKNO-1 cells as indicated by staining for senescence-associated β-galactosidase (SA-βGAL) after two sequential electroporations with the indicated siRNAs. Top panels, stained cells; bottom panel, quantitation of SA-βGAL^+^ cell numbers. n = 2 technical replicates; mean ± range. Standard bar, 50 μm. (H) Immunoblots showing the effect of RUNX1/ETO and CCND2 knockdown in Kasumi-1 and SKNO-1 cells on phosphorylation of RB1. Numbers indicate fold changes. (I) Cell cycle distribution of Kasumi-1 cells after 5 days with and without dnFOS induction by doxycycline. Ctrl, empty vector control; dnFOS, dnFOS vector-containing cells. Mean ± SD; n = 3. ^∗∗∗^p < 0.001 compared with no dox. (J) Impact of dnFOS inductions on cell doubling times (t_D_). Mean ± SD; n = 3. ^∗∗∗^p < 0.001 compared with no dox. (K) Impact of dnFOS induction on clonogenicity of Kasumi-1 cells. Colonies were counted 12 days post plating relative to no dox. Mean ± SD; n = 3. ^∗^p < 0.05 compared with no dox. See also Figure S4.

Knockdown of RUNX1/ETO impairs engraftment, proliferation, and clonal expansion and causes an accumulation of cells in the G1 phase of the cell cycle (Martinez et al., 2004, Martinez Soria et al., 2009). To examine the significance of CCND2 in these processes, we performed competitive proliferation and transplantation assays using two validated CCND2 shRNAs (Figures 4A, S4A, and S4B). Both RUNX1/ETO (shRE) and CCND2 (shCCND2-1 and -3) shRNA-expressing cells were outcompeted by control cells (shNTC) within 15 days of culture (Figures 4B and S4C). Competitive transplantation of the Kasumi-1 cells into immunodeficient *Rag2*^*−/−*^*Il2rg*^*−/−*^129×Balb/c (RG) mice resulted in a loss of cells expressing RUNX1/ETO and CCND2 shRNAs, indicating a requirement of both RUNX1/ETO and CCND2 for engraftment (Figures 4C and S4D).

As with RUNX1/ETO, depletion of CCND2 inhibited cell proliferation and clonogenic capacity and caused a G0/G1 arrest without substantially increasing apoptosis (Figures 4D–4F, S4E, and S4F). Induction of cellular senescence indicated a permanent cell-cycle arrest (Figure 4G). However, in contrast to RUNX1/ETO knockdown, neither CCND2 knockdown nor pharmacologic inhibition of CDK4/6-CCND complexes affected *CD34*, *CD33*, or *ITGAM* (CD11b) transcript levels, suggesting it did not relieve the RUNX1/ETO-mediated myeloid differentiation block (Figure S4G). Both CCND2 and RUNX1/ETO depletion led to reduced RB1 phosphorylation at serine 780 and threonine 821 (Figure 4H), sites phosphorylated by CDK4 and CDK6 (Harbour et al., 1999). Finally, interfering with AP-1 function by expression of dnFOS and the subsequent downregulation of CCND2 caused a G1 cell-cycle arrest, increased cell-doubling time, and reduced clonogenicity (Figures 4I–4K), but did not affect cell survival (Figure S4H). Induction of dnFOS did not increase the G0 fraction, and termination of dnFOS expression restored the cell-cycle distribution, suggesting that CCND2 depletion does not cause quiescence (Figure S4I). In summary, these data demonstrate that RUNX1/ETO drives leukemic proliferation and cell-cycle progression by maintaining *CCND2* expression.

### Key Regulators of G1 Progression Are Controlled by RUNX1/ETO but Do Not Compensate for CCND2 Loss

**Figure 5:**
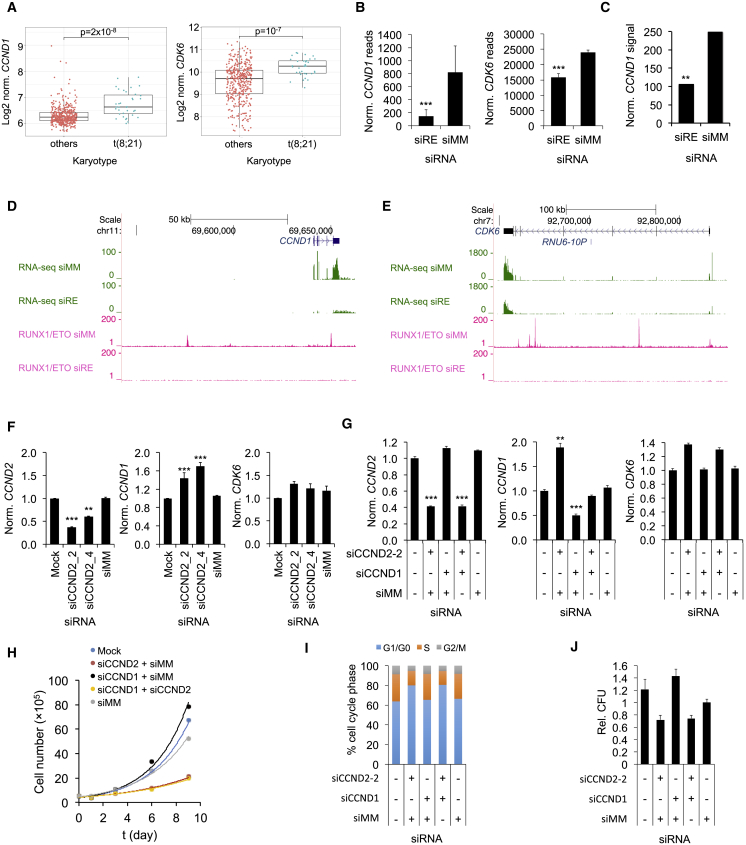
G1 Cell Cycle Components Are Regulated by RUNX1/ETO but Do Not Compensate for CCND2 Loss (A) Comparison of *CCND1* and *CDK6* expression between patients with t(8;21)-positive and -negative AML. Line, median; horizontal box, interquartile range; whiskers, 1.5× interquartile range. p value was determined by Mann-Whitney U test. Data were obtained from GEO GSE6891. (B) *CCND1* and *CDK6* expression with and without RUNX1/ETO knockdown in Kasumi-1 cells as indicated by RNA-seq. siRE, RUNX1/ETO siRNA; siMM, mismatch control siRNA. Mean ± SD; n = 3. ^∗∗∗^p < 0.001. (C) *CCND1* transcript levels in primary t(8;21) AML blasts upon RUNX1/ETO knockdown as analyzed by Illumina bead arrays with probe ILMN_1688480. ^∗∗^p < 0.01 compared with siMM. (D and E) RUNX1/ETO knockdown-induced changes in transcript levels and RUNX1/ETO binding at the *CCND1* (D) and *CDK6* (E) loci in Kasumi-1 cells as indicated by RNA-seq (green) and ChIP-seq (pink), respectively. Top, scale and base pair position on chromosome. (F) Impact of CCND2 knockdown by two different siRNAs on indicated mRNA levels. Kasumi-1 cells were sequentially electroporated every 2 days with the indicated siRNAs. Transcript levels were determined on day 8 by qPCR. Mock, non-siRNA electroporated cells; siCCND1, siCCND2, CCND1, and CCND2 siRNA. Mean ± SD; n = 3. ^∗∗∗^p < 0.001; ^∗∗^p < 0.01 compared with siMM. (G) Effects of single and combined siRNA treatment on *CCND2*, *CCND1*, and *CDK6* RNA levels in Kasumi-1 cells. Transcript levels were analyzed in triplicates 48 hr after electroporation by qPCR and normalized to *GAPDH*. Mean ± SD, n = 3. ^∗∗∗^p < 0.001; ^∗∗^p < 0.01 compared with siMM. (H) Proliferation curves for Kasumi-1 cells electroporated sequentially every 2 days with the indicated siRNA combinations. (I) Cell cycle distribution of Kasumi-1 cells 48 hr after electroporation with the indicated shRNA combinations. n = 1. (J) Colony formation of Kasumi-1 cells electroporated with the indicated sRNA combinations. Colonies were counted after 12 days post plating and normalized to siMM. Mean ± SD; n = 4 technical replicates. See also Figure S5.

We next examined whether knockdown of either RUNX1/ETO or CCND2 affected the expression of other D cyclins and G1 CDKs. While *CDK4* was not differentially expressed, *CDK6* and *CCND1* levels were significantly higher and *CCND3* levels were lower in t(8;21) AML cells compared with other AMLs (Figures 5A and S5A). RUNX1/ETO knockdown reduced *CCND1* and *CDK6* expression, with *CCND1* transcript levels also being diminished in primary AML cells (Figures 5B, 5C, and S5B–S5D). Both *CCND1* and *CDK6* loci contain several binding sites and are potential direct target genes for RUNX1/ETO (Figures 5D, 5E, and S5E–S5G).

Since loss of a single D cyclin can be compensated by increased expression of other D cyclins (Ciemerych et al., 2002, Lam et al., 2000), we determined the transcript levels of *CDK4*, *CDK6*, *CCND3*, and *CCND1* after CCND2 knockdown. Only *CCND1* transcript levels rose more than 1.5-fold while the expression of the other genes did not change (Figures 5F and S5H). However, simultaneous knockdown of both CCND1 and CCND2 did not enhance the effects of CCND2 knockdown alone (Figures 5G–5J). In conclusion, loss of CCND2 is not functionally compensated by changed expression of other components of G1 CDK-CCND complexes.

### Pharmacological Inhibition of CDK4/6-CCND Complexes Inhibits RUNX1/ETO-Driven AML

**Figure 6:**
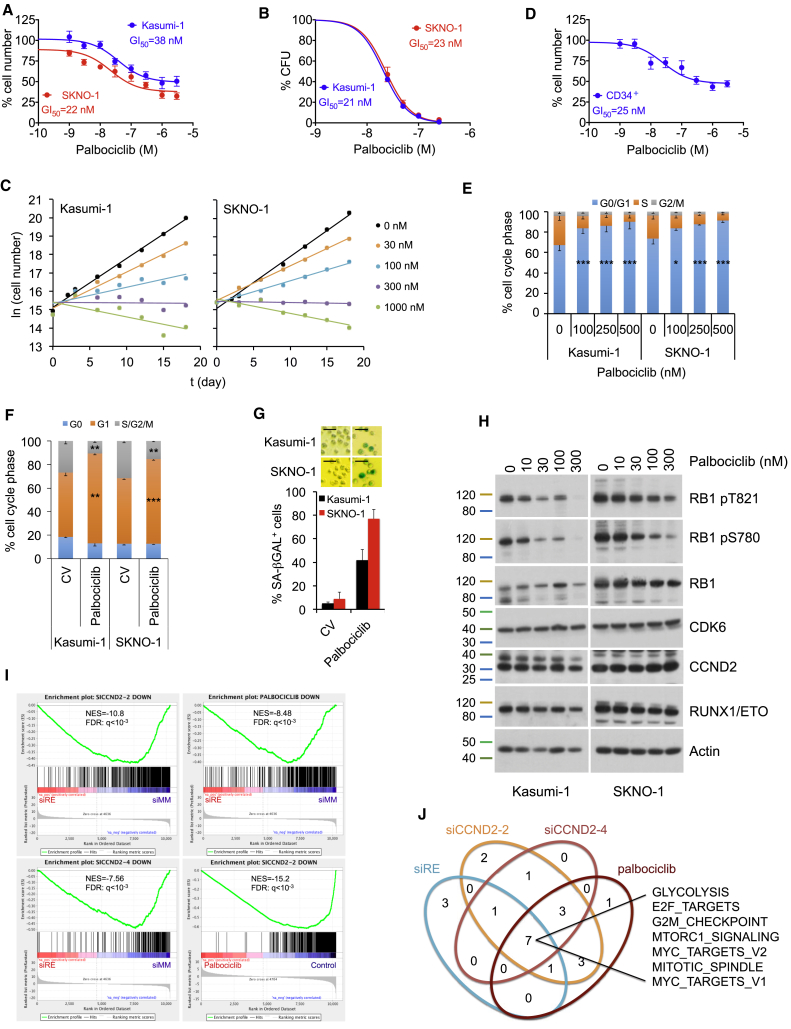
The CDK4/6 Inhibitor Palbociclib Inhibits Growth of RUNX1/ETO-Expressing Leukemic Cells (A) Dose-response curve for proliferation of SKNO-1 and Kasumi-1 cells treated for 72 hr with the indicated palbociclib concentrations. Mean ± SD; n = 6. (B) Dose-response curve for inhibition of colony formation by palbociclib. Colonies formed in presence of palbociclib were counted 14 days after seeding. Mean ± SD; n = 3. (C) Growth curves of t(8;21) cell lines during long-term treatment with palbociclib. Mean ± SD; n = 3. (D) Dose-response curve for proliferation of CD34+ cord blood cells expressing truncated RUNX1/ETO9a treated with palbociclib for 72 hr. Mean ± SD; n = 3. (E) Cell cycle distribution of Kasumi-1 and SKNO-1 cells after 72 hr treatment with the indicated palbociclib doses. Mean ± SD; n = 3. ^∗∗∗^p < 0.001; ^∗^p < 0.05 compared with no palbociclib (CV). (F) Cell cycle distribution as indicated by Pyronin Y and Hoechst33342 staining of Kasumi-1 and SKNO-1 cells with and without 50 nM palbociclib for 24 hr. ^∗∗∗^p < 0.001; ^∗∗^p < 0.01 compared with no palbociclib (CV). (G) Impact of CDK4/6 inhibition on senescence in Kasumi-1 and SKNO-1 cells as indicated by staining for SA-βGAL. Top panels, stained cells after 7 days with 50 nM palbociclib; bottom panel, quantitation of SA-βGAL-positive cell numbers. Mean ± SD; n = 5 technical replicates. Scale bar, 50 μm. (H) Immunoblots showing dose-dependent impact of 72 hr palbociclib treatment on indicated protein levels in Kasumi-1 and SKNO-1 cells. (I) GSEA for correlation between palbociclib, RUNX1/ETO, and CCND2 knockdown signatures derived from RNA-seq. NES, normalized enrichment score. (J) Hallmarks of cancer pathways shared between palbociclib treatment, CCND2, and RUNX1/ETO knockdown. Enriched pathways were identified by GSEA. See also Figure S6.

Since CCND2 binds to CDK4 and CDK6 (Matsushime et al., 1992, Meyerson and Harlow, 1994, Xiong et al., 1992), we explored whether RUNX1/ETO-expressing cells were sensitive to the CDK4/6 inhibitor palbociclib (PD-0332991) similar to MLL-rearranged (MLLr) and FLT3-ITD-positive leukemia (Placke et al., 2014, Uras et al., 2016, van der Linden et al., 2015). t(8;21) AML cell proliferation and clonogenic potential were highly sensitive to palbociclib with GI_50_ values (concentration of drug to cause 50% reduction in proliferation of cancer cells) below 50 nM and did not resume proliferation during 18 days of drug exposure (Figures 6A–6C). This sensitivity toward palbociclib is notable given that both lines carry p53 mutations (Banker et al., 1998, Matozaki et al., 1995). GI_50_ values for t(8;21)-negative AML lines varied between 60 and 230 nM (Figure S6A), and these cells also did not resume proliferation during prolonged drug exposure (Figure S6B).

To examine the effect of treatment in pre-leukemic cells, we examined the impact of pharmacological inhibition of CDK4/6 in a human pre-leukemic cell culture model expressing a truncated variant of RUNX1/ETO and a constitutive active KIT N822K receptor (Wichmann et al., 2015). Palbociclib treatment impaired proliferation (GI_50_ = 25 nM, Figure 6D), demonstrating that primary RUNX1/ETO-positive cells are also dependent on catalytically active CDK4 or CDK6.

In general, growth inhibition was primarily cytostatic for nanomolar concentrations of palbociclib with minor increases in apoptotic cells; impaired growth was associated with a dose-dependent cell-cycle arrest in the G0/G1 phase (Figures 6E and S6C). Extended treatment did not reduce G1 phase arrest (Figure S6D), emphasizing that prolonged exposure to palbociclib might not cause resistance in RUNX1/ETO-expressing cells. Moreover, it affected neither expression of myeloid differentiation markers nor the size of the G0 population (Figures 6F and S4G), but palbociclib caused a more than 10-fold increase in senescence-associated β-galactosidase positivity in both Kasumi-1 and SKNO-1 cells (Figure 6G). Finally, palbociclib reduced phosphorylation of RB1 without affecting CCND2 or CDK6 protein levels (Figure 6H). These data show that palbociclib promotes neither myeloid differentiation nor quiescence but induces a G1 cell-cycle arrest and senescence.

The observed dependence of RUNX1/ETO on its downstream target CCND2 was reflected by a substantial overlap between the transcriptional responses to palbociclib and knockdown of CCND2 and RUNX1/ETO. GSEA showed a significant correlation between gene sets associated with RUNX1/ETO-depleted, CCND2-depleted, and palbociclib-treated Kasumi-1 cells and with published RUNX1/ETO knockdown signatures (Figures 6I and S6E) (Dunne et al., 2006, Tonks et al., 2007). We found a substantial overlap of shared gene sets, including cell-cycle regulation, nucleotide metabolism, MYC- and MTOR-regulated programs, glucose transport, glycolysis, stemness, and pluripotency programs (Figures 6J and S6F). Notably, both CCND2 knockdown and palbociclib caused significant reduction of *EZH2* transcript levels (Figure S6G). Moreover, gene sets associated with CCND2 knockdown and palbociclib were inversely correlated with EZH2 and EDD gene expression signatures (Figure S6H). Since activation of EZH1/2 is associated with quiescence in leukemic stem cells (Fujita et al., 2018), these results suggest that palbociclib is unlikely to promote quiescence. Together, these findings demonstrate that the dependence of t(8;21) AML cells on CCND2 confers acute sensitivity to palbociclib.

### Inhibition of G1 CDK Activity Impairs *Ex Vivo* Expansion of Primary AML Cells

**Figure 7:**
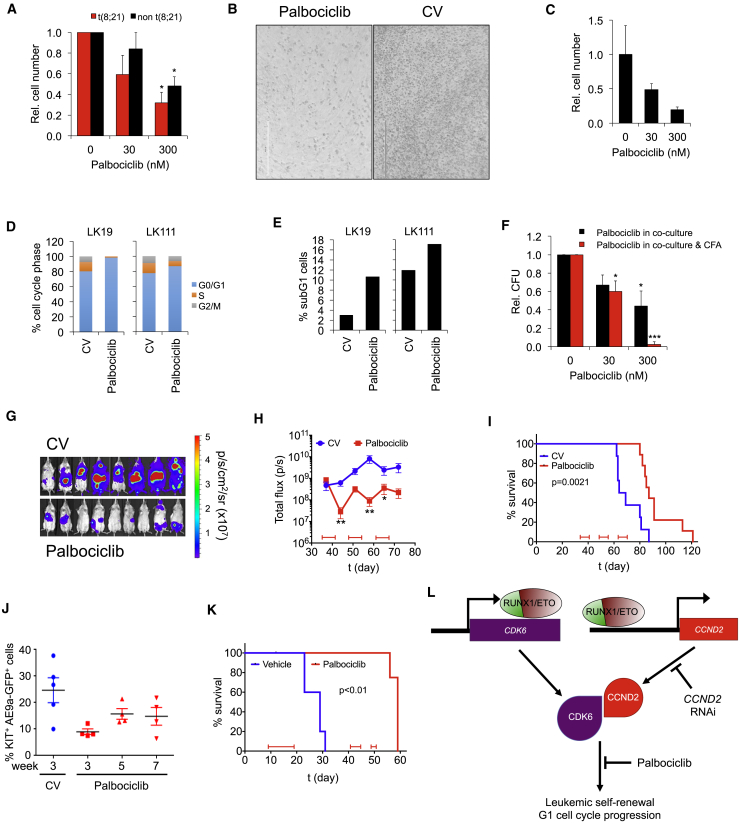
CDK4/6 Inhibition Impairs Proliferation of Primary AML Cells and Increases Median Survival *In Vivo* (A) Impact of palbociclib on proliferation of primary AML blasts. Blasts were co-cultured on MSC feeder layers with and without 300 nM palbociclib for 72 hr. Mean ± SD; n = 3 for both t(8;21) and non-t(8;21) AML patient samples. ^∗^p < 0.05 compared with no palbociclib. (B) Phase contrast photographs showing primary t(8;21) AML blasts from patient sample LK111 in co-culture with MSCs with and without (CV) palbociclib. Standard bar, 200 μm. (C) Proliferation of a t(8;21) AML sample from a relapsed patient on MS-5 feeders upon palbociclib treatment for 72 hr. Mean ± SD; n = 3 technical replicates. (D and E) Cell cycle distribution (D) and changes in apoptotic subG1 cell fractions (E) of primary AML blasts obtained from two t(8;21) patients (patient samples LK19 and LK111) on MSC after 96 hr incubation with and without (CV) palbociclib. (F) Clonogenic growth of three different t(8;21) patient samples after MSC co-culture for 96 hr. Indicated palbociclib concentrations were added either only to co-culture medium (Palbociclib in co-culture) or to both co-culture and semisolid medium (Palbociclib in co-culture & CFA). Colony numbers are relative to no palbociclib. Mean ± SD; n = 3. ^∗∗∗^p < 0.001; ^∗^p < 0.05 compared with no palbociclib. (G) Bioluminescent images of RG mice transplanted with luciferase-expressing (luc^+^) Kasumi-1 cells after 21 days of treatment with control vehicle (CV) or palbociclib. (H) Luminal flux of bioluminescence for CV (n = 8) or palbociclib-treated animals (n = 9). Treatment blocks are indicated at the bottom of the graph. ^∗∗^p < 0.01; ^∗^p < 0.05 compared with no palbociclib by one-way ANOVA using a D'Agostino and Pearson test; mean ± SEM. (I) Survival curve for RG mice transplanted with luc^+^ Kasumi-1 cells. Significance was tested by log rank test. (J) Percentage of Kit^+^ RUNX1/ETO9a (AE9a) GFP^+^ cells in mice after CV or palbociclib treatment as determined by fluorescence-activated cell sorting (FACS). (K) Survival curve for Bl6 mice transplanted with KIT^+^ AE9a GFP^+^ cells. Significance was tested by log rank test. n = 5 for both groups with one censored animal in the palbociclib group due to death unrelated to palbociclib treatment. (L) Model depicting the RUNX1/ETO-promoted G1 cell cycle progression and leukemic propagation by direct transcriptional activation of *CCND2* and *CDK6*, which can be blocked by either CCND2 knockdown or by pharmacologic inhibition of CDK4/6-CCND complexes. See also Figure S7.

Next, we examined the sensitivity of primary AML cells to palbociclib. We cultured cells obtained from t(8;21)-positive and -negative AML patients on human bone marrow-derived mesenchymal stem cells (MSCs) or, in the case of a relapsed sample, on murine MS-5 feeder layers, which support proliferation of primary AML cells (Figure S7A) (Griessinger et al., 2014, Pal et al., 2016). Importantly, palbociclib did not affect feeder cell numbers (Figure S7B). Consistent with our cell line data, palbociclib dose-dependently inhibited proliferation of primary AML blasts. A clinically achievable concentration of 300 nM palbociclib (Tamura et al., 2016) resulted in a 3-fold reduction of t(8;21)-positive AML blasts (Figures 7A, 7B, and S7C). We also tested palbociclib on a t(8;21) AML sample from a relapse patient and observed high drug sensitivity with a 5-fold reduced cell number upon treatment with 300 nM palbociclib (Figure 7C). Primary t(8;21)-negative AML cells showed an overall trend of being less sensitive to palbociclib compared with t(8;21)-positive AML (Figures 7A and S7D), although there was some heterogeneity in response concomitant with reported sensitivity in other AML subtypes (Placke et al., 2014, Uras et al., 2016, van der Linden et al., 2015). Notably, palbociclib sensitivity did not strictly correlate with cell expansion, possibly due to proliferation masked by cell-death-associated loss of cells.

Treated AML cells showed an accumulation in the G0/G1 phase and a slight increase in apoptotic cells (Figures 7D and 7E) as well as a 2-fold impediment of colony formation potential, which was further reduced to more than 10-fold when palbociclib was included in the semisolid medium (Figures 7F, S7E, and S7F). In conclusion, palbociclib impairs both the expansion and clonogenicity of primary AML blasts.

### Palbociclib Inhibits Leukemia *In Vivo*

To confirm that CCND2 was required to propagate t(8;21) *in vivo*, we examined the efficacy of palbociclib in RG mice transplanted with Kasumi-1 cells by initiating treatment and following the development of disseminated luciferase signal (Figures 7G, 7H, and S7G). Pharmacokinetic analysis indicated that a single oral application of 100 mg/kg palbociclib achieved plasma levels in the micromolar range over a period of 24 hr (Figure S7H), thus exceeding the plasma levels reported for breast cancer patients (Tamura et al., 2016). Palbociclib treatment halted disease progression and induced a transient decrease in luciferase signal (Figure 7H), and overall survival in palbociclib-treated mice was significantly longer (87 days) than control mice (67 days) (Figure 7I).

To extend these studies to an *in vivo* setting that represents the AML hierarchy and clonal variety of primary AML, we tested the anti-leukemic effect of palbociclib in a mouse leukemia model expressing a C-terminally truncated isoform of RUNX1/ETO, termed RUNX1/ETO9a (Draper et al., 2016, Yan et al., 2006). ChIP experiments with RUNX1/ETO9a in KIT-positive hematopoietic stem and progenitor cells showed binding of the truncated protein to the corresponding murine *Ccnd2* and *Cdk6* cis-elements as shown for the full-length RUNX1/ETO protein in human cells (Figures S7I and S7J). Consistently, palbociclib substantially reduced the leukemic burden, delayed AML progression, and increased the median survival from 29 days in the control group to 59 days (Figures 7J, 7K, and S7K). Taken together, the RUNX1/ETO-supported expression of drivers of G1 cell-cycle progression is causatively linked with strong single-agent activity of palbociclib against t(8;21) AML *in vitro* and *in vivo* (Figure 7L).

### Inhibition of G1 CDK Activity Sensitizes AML Cells toward KIT Inhibition

**Figure 8:**
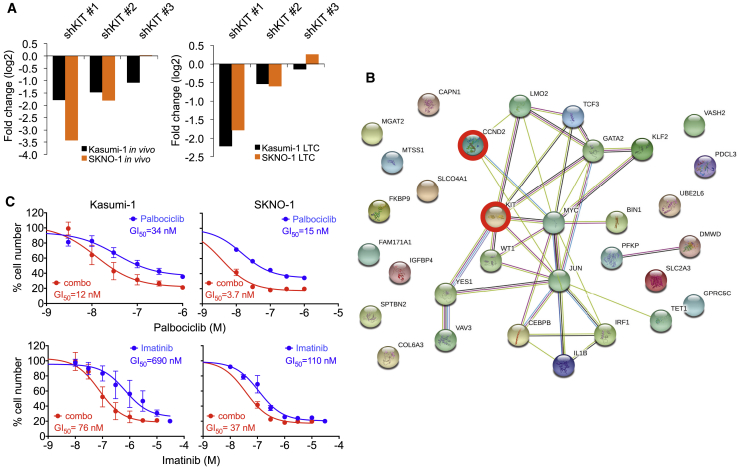
CDK4/6 Interference Sensitizes AML Cells toward Inhibition of Mutated KIT (A) Fold change of all KIT shRNA constructs in RNAi screens after third replatings (top) and *in vivo* engraftment (bottom) in t(8;21) cell lines. (B) String-generated gene network showing interactions between genes indicated by the *in vivo* RNAi screen. Nodes represent genes indicated by at least two shRNAs in combined SKNO-1 and Kasumi-1 screens. (C) Dose-response curves for proliferation of Kasumi-1 and SKNO-1 cells with palbociclib, imatinib (blue curves), or a combination with a fixed molar ratio of palboclib:imatinib of 1:10 (red curves). Top and bottom x axes show the corresponding palbociclib and imatinib concentrations. Cell numbers were counted after 72 hr of drug treatment. Mean ± SD; n = 4. See also Figure S8.

Finally, we asked whether interference with G1 CDK activity would create therapeutic vulnerabilities. Activating KIT mutations are among the most frequent secondary mutations found in t(8;21) AML and indicate poor clinical outcome (Wang et al., 2005, Wichmann et al., 2015). Since both Kasumi-1 and SKNO-1 express KIT^N822K^, it was interesting to note that KIT shRNAs were depleted in both of the RNAi screens (Figure 8A), emphasizing the significance of KIT mutants in t(8;21) AML propagation (Becker et al., 2008, Faber et al., 2016, Larizza et al., 2005). STRING network analysis of genes indicated in the screen by at least two shRNAs showed no direct interaction between KIT and CCND2. However, KIT may affect CCND2 indirectly via MYC and JUN/AP-1 and may also regulate pathways such as MTOR that are targeted by RUNX1/ETO (Figures 8B and S8A) (Rossi et al., 2006, Serve et al., 1995). Previous studies demonstrated responsiveness of KIT^N822K^-expressing t(8;21) cells to imatinib, an inhibitor of ABL, BCR/ABL, PDGF receptor, and KIT (Wang et al., 2005). Palbociclib was synergistic with imatinib in Kasumi-1 and SKNO-1 cells with combination indices below 0.6 (Figures 8C and S8B). Therefore, interference of RUNX1/ETO-driven G1 progression sensitizes leukemic cells to inhibition of mutated KIT, a major secondary event in t(8;21) AML. Since RUNX1/ETO and KIT^N822K^ represent initiating and secondary events of leukemogenesis, these data suggest that concurrent targeting of the two mutations may offer substantial therapeutic benefit.

## Discussion

The molecular mechanisms by which RUNX1/ETO promotes *CCND2* expression highlights the complexity of how leukemogenic transcription factors reprogram the epigenome and establish an aberrant transcriptional network essential for leukemia maintenance and self-renewal. RUNX1/ETO has been described to activate gene expression by interacting with the histone acetylase EP300 (Wang et al., 2011). However, here we show that RUNX1/ETO can also activate gene expression by interfering with an intergenic negative regulatory element. Members of the RUNX family, including RUNX1, have been previously reported to interact with silencing elements to restrict gene expression to distinct cell lineages (Setoguchi et al., 2008). The activation of *CCND2* transcription by RUNX1/ETO binding to the −30 kb region may represent another example for the interaction of RUNX proteins with a negative regulatory element.

However, RUNX1/ETO does not only directly regulate *CCND2* expression. AP-1 activity is required to drive *CCND2* expression and cell growth, and RUNX1/ETO loss reduces expression of AP-1 family members, which affects the recruitment of AP-1 factors to the *CCND2* promoter (Mathas et al., 2002). RUNX1/ETO is thought to promote JUN expression by activating JNK signaling (Elsasser et al., 2003, Frank et al., 1999). Our ChIP-seq data show RUNX1/ETO occupancy at *JUN* and all three members of the FOS gene family, suggesting also direct regulation (Ptasinska et al., 2014). Therefore, RUNX1/ETO activates *CCND2* transcription both directly and indirectly.

Approximately 10%–15% of all t(8;21) AMLs have been found to harbor *CCND2* mutations (Eisfeld et al., 2017, Faber et al., 2016). All mutations are located within the C-terminal PEST domain of CCND2, increasing its stability and activity and further highlighting its relevance for maintaining t(8;21) AML. Importantly, leukemic cells expressing mutant CCND2 remain sensitive to CDK4/6 inhibitors such as palbociclib (Khanna et al., 2017).

Similar to previous preclinical studies, we used palbociclib doses of 100–150 mg/kg in our *in vivo* experiments (Fry et al., 2004), which yielded 10-fold higher plasma levels than those found in breast cancer patients treated with palbociclib (Tamura et al., 2016). Future experiments will refine the precise palbociclib dose that, as single agent or in combination, will inhibit t(8;21) AML propagation.

In conclusion, we identified *CCND2* as a core component of the RUNX1/ETO-driven AML program. This dependence translates into a high susceptibility toward CDK4/6 inhibition, which could be employed in combination with agents targeting secondary mutational events such as KIT^N822K^, thus offering alternative therapeutic options. Ongoing research will identify drug combinations based on synergistic activity with CDK4/6 inhibitors for more precise intervention without occurrence of treatment resistance and long-term side effects.

## STAR★Methods

### Key Resources Table

**Table:** 

REAGENT or RESOURCE	SOURCE	IDENTIFIER
**Antibodies**		

Rabbit anti-phospho Rb (Ser780)	Cell Signaling Technology	Cat#9307; RRID: AB_330015
Rabbit anti-phospho Rb (Thr 826)	Abcam	Cat#ab133446; RRID: AB_2722666
Rabbit anti-phospho Rb (Thr821)	Abcam	Cat#ab4787; RRID: AB_304264
Mouse anti-CCND2	Proteintech	Cat#10934-1-AP; RRID: AB_2275319
Mouse anti-Rb	BD Pharmingen	Cat#554136; RRID: AB_395259
Rabbit anti- AML1/RHD Domain (50-177)	Millipore	Cat#PC285; RRID: AB_213572
Rabbit anti-AML1	Cell Signaling Technology	Cat#4334; RRID: AB_2184099
Mouse anti-Clathrin Heavy Chain	BD Biosciences	Cat#610500; RRID: AB_397866
Mouse anti-β-Actin (HRP conjugated)	Abcam	Cat#ab49900; RRID: AB_867494
Rabbit anti-CDK6	Cell Signaling Technology	Cat#13331; RRID: AB_2721897
Mouse anti-glyceraldehyde-3-phosphate dehydrogenase (GAPDH) (Clone 6C5)	Hytest	Cat#5G4-6C5; RRID: 1616722
Goat anti-rabbit immunoglobulins antibody	Dako	Cat#P0448; RRID: AB_2617138
Goat anti-mouse immunoglobulins antibody	Dako	Cat#P0447; RRID: AB_2617137

**Bacterial and Virus Strains**		

One Shot STBL3 chemically competent *E. coli*	Invitrogen	Cat#C7373-03

**Chemicals, Peptides, and Recombinant Proteins**		

Palbociclib Isethionate (PD-0332991) (*in vivo*)	DC Chemicals	Cat#DC8470
Palbociclib (*in vitro*)	DC Chemcials	Cat#DC5067
Vorinostat (Suberoylanilide hydroxamic acid)	Sigma	Cat#SML0061
Imatinib (STI571)	Selleckchem	Cat#S2475

**Critical Commercial Assays**		

UltraClean Endotoxin-Free Mini plasmid Prep Kit	Mo Bio Laboratories	Cat#12311-100
Qiaprep Spin Miniprep Kit	Qiagen	Cat#27106
Qiaquick Gel Extraction Kit	Qiagen	Cat#28706
Endofree Plasmid Maxi Kit	Qiagen	Cat#12362
UltraClean 96 PCR Cleanup Kit	Qiagen	Cat#12596-4
RNAeasy Mini Kit	Qiagen	Cat#74106
Qiashredder	Qiagen	Cat#79656
RevertAid First Strand cDNA Synthesis Kit	Thermoscientific	Cat#K1622
DNeasy Blood & Tissue Kit	Qiagen	Cat#69506
Bio-Rad Protein Assay Dye Reagent Concentrate	BioRad	Cat#500-0006
Pierce BCA Protein Assay Kit	ThermoScientific	Cat#23227

**Deposited Data**		

Raw Bead Array, ChIP-seq, DHS-seq data	(Ptasinska et al., 2012)	GEO: GSE29225
Raw RNA-seq, Chip-seq, DHS-seq data	(Ptasinska et al., 2014)	GEO: GSE60121
Raw ChIP-seq (JUND) and CHiC data	This paper	GEO: GSE117108

**Experimental Models: Cell Lines**		

Kasumi-1	DSMZ	RRID: CVCL_0589 Cat#ACC 220
Kasumi-1 pSLIEW	In house (Bomken et al., 2013)	N/A
SKNO-1	DSMZ	Cat#ACC 690; RRID: CVCL_2196
SKNO-1 pSLIEW	In house (Bomken et al., 2013)	N/A
HL-60	DSMZ	Cat#ACC 3; RRID: CVCL_0002
AML-3	DSMZ	Cat#ACC 582; RRID: CVCL_1844 Cat#ACC 582
THP-1	DSMZ	Cat #ACC 16; RRID: CVCL_0006
MV4-11	DSMZ	Cat#ACC 102; RRID: CVCL_0064
HEK293T	DSMZ	Cat#ACC 305; RRID: CVCL_0063
Human bone marrow-derived mesenchymal stromal cells (MSC)	In house (Pal et al., 2016)	N/A
MS-5	DSMZ	Cat# ACC-441; RRID: CVCL_2128
Human peripheral blood CD34^+^ RUNX1/ETO^+^ KIT N822K HSPCs	Wichmann et al., 2015	N/A
AML patient samples, see Table S4	N/A	N/A

**Experimental Models: Organisms/Strains**		

*NOD.Cg-Prkdc*^*scid*^*Il2rg*^*tm1Wjl*^*/SzJ* (NSG) mice	Jackson Laboratory	N/A
*Rag2*^*−/−*^*Il2rg*^*−/−*^*129×Balb/c* (RG) mice	Jackson Laboratory	N/A
C57BL/6 *AML1-ETO9a-IRES-GFP::rtTA* mice	In house (Draper et al., 2016)	N/A
C57BL/6 Trp53+/- mice	In house (Donehower et al., 1992)	N/A
C57BL/6 *AML1-ETO9a-IRES-GFP+::rtTA+::Trp53+/-* mice	In house (Draper et al., 2016)	N/A
C57BL/6 mice	Envigo	N/A

**Oligonucleotides**		

siRE (sense, 5’-CCU CGA AAU CGU ACU GAG AAG-3’, antisense, 5’ UCU CAG UAC GAU UUC GAG GUU-3’),	In house (Heidenreich et al., 2003)	N/A
siMM (sense, 5’-CCU CGA AUU CGU UCU GAG AAG-3’; antisense, 5’-UCU CAG AAC GAA UUC GAG GUU-3’),	In house (Heidenreich et al., 2003)	N/A
CCND2 siRNA	Qiagen	Hs_CCND2_2 FlexiTube SI00027839 and Hs_CCND2_4 FlexiTube SI00027853
CCND1 siRNA	Qiagen	(Hs_CCND1_3 FlexiTube SI00147826)
Decode Indexing PCR and Sequencing Primer Kit	Dharmacon	#RHS5339 Sequences upon request from Company
qPCR primer sequences, see Table S5	N/A	N/A

**Recombinant DNA**		

pSLIEW vector	In house (Bomken et al., 2013)	N/A
pCMVΔ8.91 packaging vector	Life Science Market	Cat# PVT2323
pMD2.G envelope vector	Addgene	Cat#12259
pTRIPZ lentiviral vector	Dharmacon (ThermoScientific)	Cat#RHS4750
pLKO5d.SFFV.miRNA30n	Addgene	Cat#90333
pCW57.1-dnFOS	In house	N/A

**Software and Algorithms**		

Bowtie2	Langmead and Salzberg, 2012	http://bowtie-bio.sourceforge.net/bowtie2/index.shtml
EdgeR	Robinson et al., 2010	http://bioconductor.org/packages/edgeR
Limma/Voom	Law et al., 2014	http://bioconductor.org/packages/limma
RUVSeq	Risso et al., 2014	http://bioconductor.org/packages/RUVSeq
STAR	Dobin et al., 2013	https://github.com/alexdobin/STAR
HTseq	Anders et al., 2015	https://github.com/simon-anders/htseq
DESeq2	Love et al., 2014	http://bioconductor.org/packages/DESeq2
MAGeCK	Li et al., 2014	https://sourceforge.net/projects/mageck/
GEOquery	Davis and Meltzer, 2007	http://bioconductor.org/packages/GEOquery
affy	Irizarry et al., 2003	http://bioconductor.org/packages/affy
ggplot2	CRAN	https://CRAN.R-project.org/package=ggplot2
GSEA	Subramanian et al., 2005	http://software.broadinstitute.org/gsea
SynergyFinder	Ianevski et al., 2017	https://synergyfinder.fimm.fi

### Contact for Reagent and Resource Sharing

Further information and requests for reagents and resources should be directed to and will be fulfilled by the lead contact Olaf Heidenreich (olaf.heidenreich@ncl.ac.uk).

### Experimental Model and Subject Details

#### Cell Lines

Kasumi-1 (RRID: CVCL_0589; male), SKNO-1 (RRID: CVCL_2196; male). Kasumi-1 C28 and SKNO-1 C10 are subclones of Kasumi-1 and SKNO-1 respectively and contain the pSLIEW vector allowing cells to express firefly luciferase and eGFP (Bomken et al., 2013). Cells were maintained in RPMI1640 medium supplemented with 10% or 20% FBS respectively, the latter also supplemented with GM-CSF (7ng/ml)] at 37°C in a humidified 5% CO_2_ incubator. Neither Kasumi-1 nor SKNO-1 contain CCND2 mutations according to CCLE (https://portals.broadinstitute.org/ccle) and COSMIC (http://cancer.sanger.ac.uk/cell_lines) databases.

Human peripheral blood CD34^+^ RUNX1/ETO^+^ KIT N822K HSPCs (Wichmann et al., 2015) were cultured in IMDM supplemented with 20% FBS, 2% Glutamine, 1% Penicillin/Streptomycin and the following human cytokines at the indicated final concentrations: IL3 (10 ng/ml), IL6 (20 ng/ml), FLT3L (20 ng/ml), GM-CSF (7 ng/ml), SCF (20 ng/ml) and TPO (20 ng/ml) 37°C in a humidified 5% CO_2_ incubator.

HL-60 (RRID: CVCL_0002; female), AML-3 (RRID: CVCL_1844; male), THP-1 (RRID: CVCL_0006; male), and MV4-11 (RRID: CVCL_0064; male) were maintained in RPMI1640 medium supplemented with 20% FBS at 37°C in a humidified 5% CO_2_ incubator. For shRNA knockdown cells, the culture medium was additionally supplemented with 2 μg/ml puromycin.

Human bone marrow-derived mesenchymal stromal cells (MSC) were isolated as described previously (Pal et al., 2016). MSC were maintained in DMEM supplemented with 20% FBS, 1% Penicillin/Streptomycin, 1% Glutamine, FGF-1 (8 ng/ml). MS-5 (RRID: CVCL_2128, murine) were maintained in α-MEM supplemented with 10% FBS.

HEK293T cells (RRID: CVCL_0063; female) for lentivirus production were maintained in HEPES-modified DMEM medium supplemented with 10% FBS, 4mM L-glutamine and 1mM sodium pyruvate, incubated as above.

Identity of cell lines was confirmed by short tandem repeat profiling by NewGene Ltd (Newcastle University, UK). Cell lines were confirmed free from mycoplasma infection at regular intervals using a MycoAlert kit (Lonza, Slough, UK).

#### Primary Cultures

Patient-derived AML blasts were obtained from the Newcastle Haematology Biobank (REC reference number 07/H0906/109+5) and were cultivated on MSC or MS-5 layers in SFEMII supplemented with StemSpan™ Myeloid Expansion Supplement, IL3 (10 ng/ml), FLT3L (20 ng/ml), 20% FBS and 1% glutamine or Myelocult H5100 supplemented with StemSpan™ Myeloid Expansion Supplement, Hydrocortisone, IL3, FLT3L and glutamine. Patient details are given in Table S4.

#### *In Vivo* Mouse Studies

Mice for the RNAi screen and those for the intrahepatic *in vivo* palbociclib treatment model were housed in the Comparative Biology Centre (Newcastle University) under specific pathogen free conditions. All experimental manipulations were performed under sterile conditions in a laminar flow hood, except imaging. All work was approved and conductedn accordance with Home Office Project Licences PPL60/4552 and PPL60/4222 by researchers who had completed approved Home Office training and held current Personal Licences under the Animals (Scientific Procedures) Act 1986. Mouse studies were approved and conducted at Newcastle University and University of Manchester following Institutional ethical review (AWERB) and in accordance with the UK Home Office Animals (Scientific Procedures) Act 1986. Group sizes were chosen according to pilot experiments and male and female mice randomly assigned into those groups

*NOD.Cg-Prkdc*^*scid*^ *Il2rg*^*tm1Wjl*^*/SzJ* (NSG) mice (male and female) aged between 10 and 15 weeks at study commencement were used for the intra-femoral RNAi screen *in vivo* model. Dependent on cell line injected (Kasumi-1 or SKNO-1), mice were randomly assigned to 2 treatment groups: 13-14 mice fed a doxycycline-free diet and 6-7 mice fed a Doxycycline diet. After 4 weeks, the Doxycycline-free group was further randomly divided into 2 groups of 6-7 mice: a Doxycycline free-group and “Doxycycline after engraftment” (dox delayed) group.

*Rag2*^*−/−*^*Il2rg*^*−/−*^129×Balb/c (RG) mice (male and female) aged 1-4 days at study commencement were used for the intrahepatic *in vivo* palbociclib treatment model. They were then randomly assigned to 2 treatment groups (5-6 mice per group) prior to treatment. Male and female mice aged between 10 and 15 weeks at study commencement were used for intravenous transplantation.

For both study models, mice were humanely killed when they displayed end points as specified by the licenses. For example when tumours reached 1.5 cm in diameter, if they lost >10% weight compared to controls for 3 consecutive days or 20% at any time, or they displayed signs of ill health.

The *AML1-ETO9a-IRES-GFP::rtTA* mouse line has been described previously (Draper et al., 2016). These mice were crossed with the previously described C57BL/6 *Trp53+/-* (Donehower et al., 1992) generating *AML1-ETO9a-IRES-GFP+::rtTA+::Tp53+/-* mice. We utilized a *Tp53* heterozygous background as p53 loss accelerates AML1-ETO9a-mediated AML (Zuber et al., 2009). Male and female mice were intravenously transplanted at an age of 12 weeks.

C57BL/6 mice (male and female) were used for secondary transplantations at an age of 12 weeks.

### Method Details

#### Chemicals and Reagents

The following antibodies were used for western blot analysis: beta-Actin, ab49900 (Abcam); CDK6, D4S8S (Cell Signalling); Clathrin (C28, BD Biosciences); Cyclin D2, 10934-1-AP (Proteintech); Phospho-Rb (Ser780), 9307 (Cell Signalling); Phospho-Rb (Thr821), ab4787(Abcam); Rb, 554136 (BD Pharmingen); RUNX1/ETO, PC285 (Merck Millipore).

Growth factors and media were obtained from StemCell Technologies, Sigma- Aldrich, Gibco and R&D Systems. Palbociclib was purchased from DC Chemicals (Shanghai, China).

dnFOS was amplifided from cDNA provided by Charles Vinson (Olive et al., 1997) with SalI and NotI restriction site overhangs. Using these restriction sites, the fragment was ligated into pENTR2B (Addgene) and then recombined into pCW57.1 (Addgene).

#### Knockdown Using siRNA Electroporation

Kasumi-1 and SKNO-1 cells were transfected with the indicated siRNA concentrations using a Fischer EPI 3500 electroporator as described previously (Martinez et al., 2004). siRNA was added to final concentration of 100-500 nM to 100-750 μl of cell suspension (10^7^ cells/ml) in standard medium into a 0.4 cm electroporation cuvette. Electroporation was performed at 330 V (Kasumi-1) or 350 V (all other cell lines) for 10 ms. After 15 min at room temperature, cells were diluted and cultured under standard conditions. The following siRNAs were used: RUNX1/ETO siRNA siRE, mismatch control siMM, CCND2 siRNAs siCCND2-2 and siCCND2-4 and CCND1 siRNA siCCND1-3.

#### Drug Treatments

If not otherwise indicated, palbociclib (DC Chemicals, Shanghai) was used at various concentrations for 72 h before counting using Trypan Blue solution (0.4%) to count cell number. Patient-derived AML blasts were treated with palbociclib for 96 hr. Vorinostat (Suberoylanilide hydroxamic acid, Sigma Aldrich) was used at 1 μM for 24 h. Combination treatments included the use of palbociclib with imatinib (Sigma) at various concentrations for 72 h before counting cell number using Trypan Blue solution (0.4%) or assessed via luciferase assay normalised to known cell numbers. The GI_50_ is defined as the concentration required for 50% of maximal inhibition of cell proliferation. Synergy scores were calculated using SynergyFinder (Ianevski et al., 2017).

#### Lentivirus Production

Lentivirus were produced in 293T cells by co-transfection with a second generation lentiviral vector, an envelope plasmid pMD2.G and a packaging plasmid pCMVΔR8.91. The day before co-transfection, cells were seed at a density of 2-3 × 10^5^ cells/ml on a 100-mm tissue culture dish. The day of co-transfection 5 μg of pMD2.G, 15 μg of pCMVR8.91 and 20 μg the lentiviral vector were mixed and brought to a final volume of 250 μl with special water (2.5 mM HEPES containing deionized water at pH7.3). After adding 250 μl of 0.5 M CaCl_2_ solution, this mix was added dropwise, slowly on 500 μl of 2 × HeBS (0.28 M NaCl, 0.05 M HEPES and 1.5 mM Na_2_HPO_4_ in deionized water at pH 7.0), while mixing by air bubbling. The mix is left to incubate for 30-40 min at RT before adding it slowly, dropwise on the cell monolayer. After 24 h, cells were gently washed once with 10 ml prewarmed PBS, and 10 ml of fresh complete media was added. Cells were incubated at 37°C in a humidified atmosphere with 5% CO_2_ for the next 3 days. Lentivirus was collected by centrifuging the cell culture medium supernatant at 3000 rpm of 15 min at 4°C. The supernatant was subsequently filtered through Acrodisc^®^ Syringe 0.45 filters and stored in aliquots at -80°C.

#### Cell Transduction

After adding polybrene to a starting dilution of cells at a 10^6^ cells/ml to a final concentration of 8 μg/ml, cells were seeded onto a cell culture plate. Virus containing supernatant was added to the cell suspension and the plate was centrifuged at 34°C for 50 min at 900xg. After centrifugation, cells were incubated overnight. On the next day the supernatant was removed and cells were diluted 1:2 in complete media. dnFOS transduced cells were puromycin selected for 5 days followed by single cell sorting on a FACS Aria II to grow up individual clones.

#### RNAI Screen Library

A customized shRNA library was purchased from Thermo Scientific. shRNAmir constructs that were unavailable as pTRIPZ constructs were cloned from pGIPZ to pTRIPZ as indicated by the provider (Thermo Scientific Open Biosystems Expression Arrest TRIPZ Lentiviral shRNAmir technical manual) with the exceptions of using Qiaprep Spin Miniprep Kit, Qiaquick Gel, Endofree Plasmid Maxi Kit and Qiaquick Gel Extraction Kit for the cloning stages and UltraClean Endotoxin-Free Mini plasmid Prep Kit to provide endotoxin free DNA plasmids. One Shot STBL3 chemically competent *E. coli* were used for vector over-expression. The complete shRNA construct library can be found in Table S1. The screen lentiviral library provided 1000-fold coverage for each construct in both Kasumi-1 cells and SKNO-1 cells engineered to express GFP and Luciferase (Bomken et al., 2013). shNTC virus was added to the screen library lentivirus therefore making up 14% of the screen lentiviral pool. shRE containing cells were added to shRNA library containing cells to ensure equal coverage of shRE to other library shRNA in the screen (each construct covers 0.2% of the library. shNTC covers 14% of the library). For a more than 1,000-fold coverage, 10^7^ Kasumi-1 or SKNO-1 cells were transduced at an MOI of 0.3 and subsequently selected with puromycin (2 μg/ml) in the absence of doxycycline, i.e. without inducing shRNA expression prior to colony formation and xenotransplantation experiments. Cells were treated in the absence and presence of doxycycline for 3 days under continuous puromycin selection and subsequently put into culture, colony formation experiments (see Colony Formation Assays section) and xenotransplantation (see below).

#### Mouse Transplantations

For the RNAi screen, SKNO-1 or Kasumi-1 were intrafemorally injected into NOD.Cg-Prkdc^scid^ Il2rg^tm1Wjl^/SzJ (NSG) mice at a total cell number of 5x10^5^ and 1x10^5^, respectively (for mouse group details see *in vivo* mouse studies section above). We originally applied three different doxycycline schedules for inducing shRNA expression with one untreated control group (Kasumi-1: n=5; SKNO-1: n=2; no dox), one group being treated with doxycycline starting with the time point of transplantation (Kasumi-1: n=5; SKNO-1: n=3; dox) and one group with doxycycline treatment initiated 28 days after transplantation to avoid interference with homing ((Kasumi-1: n=3; dox delayed). However, PCA of shRNA pool compositions showed a clear separation of the latter two groups and the control group, but the two different doxycycline schemes did not cause a further segregation (Figure 1D). Therefore, we combined all doxycycline-treated animals into one group for further analyses. Leukemic cell propagation and location was tracked by bioluminescence using the IVIS Imaging System (Caliper). Expression of shRNA in all tumour samples was indicated by co-expression of RFP and observed on the FACS Calibur.

For primary mouse AML1-ETO9a (AE9a) transplantations, NOD.Cg-Prkdc^scid^ Il2rg^tm1Wjl^/SzJ (NSG) mice (bred from a colony supplied by the Jackson Laboratory) were fed Low-Phytoestrogen irradiated complete feed supplemented with 545 mg/kg (625 ppm) doxycycline hyclate (ssniff Spezialdiäten GmbH), commencing from seven days prior to transplantation. In addition, they were supplied with drinking water supplemented with 0.16% neomycin sulfate (ThermoFisher Scientific) for a total of sixteen days, commencing from three days prior to transplantation. On day 0, the mice were conditioned with sublethal full-body irradiation (1.25 Gy) and injected with 2x10^6^ *AE9a-IRES-GFP+::rtTA+::Tp53+/-* bone marrow cells (post-Ammoniun-Chloride-Potassium (ACK) buffer lysis). Spleen cells were harvested from primary recipient mice displaying a leukaemic phenotype (approximately 20 weeks post transplantation).

For secondary mouse AE9a transplantations, we chose C57 Bl/6 mice as the immunologically more relevant therapeutic model compared to NSG mice. C57BL/6 mice (supplied by Envigo) were fed doxycycline hyclate-supplemented complete feed and neomycin sulfate-supplemented drinking water as above. On day 0, the mice were conditioned with sublethal full-body irradiation (two doses of 3.5 Gy each, three hr apart) and then injected with 2x10^6^ AE9a-IRES-GFP-expressing spleen cells.

#### RNAI Screen Sample Collection

Samples of cells were taken throughout both screens. For the *in vivo* screens, leg and abdominal tumours formed by t(8;21) cells, were harvested from humanely killed mice. DNA was extracted using a DNeasy Blood & Tissue Kit (Qiagen). PCR was performed on each sample using the Decode Indexing PCR and Sequencing Primer Kit (Dharmacon), with 36 further bespoke reverse primers. The forward primer was adapted for sequencing the pTRIPZ vector (AAT GAT ACG GCG ACC ACC GAG ATC TAC ACG TGA TGC AGA AGA AAA CAC G). Amplicons were electrophoresed on an agarose gel, bands cut out and cleaned up using the Qiagen PCR clean-up kit. Samples were pooled into groups of 48 samples and sent for Illumina MiSeq 50bp single end (SE) sequencing and later Illumina HiSeq 50bp SE sequencing.

#### Colony Formation Assays

Cells were seeded in Methylcellulose media (0.56% (w/v) in complete media containing 20% FBS) at a density of 5,000 cells/ml on a 24 well plate (2500 cells per well), and incubated 10-14 days until colonies grew to over 25 cells/colony before counting. RNAi screen cells (4x10^6^ cells/group) were similarly diluted onto 10 cm tissue culture plates.

#### Cell Cycle Analysis

Approximately 10^6^ cells were resuspended in 200 μl of citrate buffer (0.25 M Sucrose, 40 mM Sodium citrate pH 7.6). After that, 2 μl 100 mg/ml RNase A was added followed by 800 μl of staining solution (20 μg/ml propidium iodide, 0.5% NP40, 0.5 mM EDTA in PBS at pH 7.2). Cells were acquired on FACS Calibur in the FL2-H channel. Data were subsequently analysed using FlowJo software.

#### Apoptosis Assay (Annexin V Staining)

Cells were washed twice with cold PBS and resuspended in 1× binding buffer (10X: 0.1 M Hepes (pH 7.4), 1.4 M NaCl, 25 mM CaCl_2_) at a concentration of 1 × 10^6^ cells/ml. After transfer of 100 μl of the cell suspension (10^5^ cells) to a 5 ml culture tube and addition of 5 μl of BV421 Annexin V (BD Biosciences, 563973), cells were vortexed and incubated for 15 min at RT in the dark. After addition of 400 μl of 1× Binding Buffer, cells were analysed on FACS Canto II using FlowJo software.

#### Senescence Assay (B- Galactosidase Staining)

Cytochemical staining for beta β-galactosidase activity was performed using Senescence β-Galactosidase Staining Kit (# 9860, Cell Signalling). Cells were collected and washed with 10 ml PBS. Cells were then fixed with fixative solution at room temperature for 15 minutes, followed by two PBS wash steps. Staining reagent at pH 6 was prepared according to manufacturer’s instructions and 500 μl of the solution was added to each cell pellet. Samples were then transferred into a 24 well plate, sealed with parafilm, and placed in an incubator at 37°C without CO2 overnight. The following day, stained samples were viewed under an Axiovert 200 microscope (Zeiss) at 200x magnification, with bright field illumination. A total of 5 images were captured for each sample and assessed using ImageJ image analysis software. The percentage of β-galactosidase positive undergoing senescence (stained green) were determined by counting the number of the green cells and normalising them to the total number of the cells in the same image.

#### Western Blotting and QPCR

Cells were lysed in either 50mM Tris pH 8/1% SDS supplemented with phosphatase and protease inhibitors or RIPA buffer (50 mM Tris, 150 mM NaCl, 1% Triton X-100, 1% Sodium Deoxycholate, 0.1% SDS, 1 mM EDTA) supplemented with phosphatase and protease inhibitors. Alternatively, proteins present in the RNeasy flow through were mixed with 2 volumes of acetone, precipitated and dissolved in urea buffer (8 M urea, 1% DTT, 4% CHAPS). Protein concentration was determined using either Bio-Rad Protein Assay Reagent concentrate (Bio-Rad) or BCA Protein Assay kit (Pierce). Sample proteins were separated using SDS-PAGE electrophoresis and transferred onto PVDF. Membranes were incubated overnight with primary antibody, washed and incubated with secondary antibody for 1 hr. Protein/antibody complexes were detected by autoradiography.

Total RNA was extracted using RNeasy Mini Kit (Qiagen, 74106). 500 ng was used as a template for reverse transcriptase with RevertAid First Strand cDNA Synthesis Kit (ThermoScientific, K1621). Quantitative PCR was then performed on a Viia7 RT-PCR (Applied Biosystems). Primer sequences are shown in Table S5.

#### Next Generation Sequencing

50 bp single end (SE) sequencing was performed using Illumina MiSeq and HiSeq2000. Resulting reads consisted of shRNA specific barcodes in FASTQ format. Raw reads were trimmed both ends up to the locations of barcode sequence before aligning to the reference shRNA barcodes using Bowtie2 (Langmead and Salzberg, 2012). An in-house script was used to count the number of reads specific to each shRNA barcode from the Bowtie2 output allowing for only a single mismatch. Barcode read counts were then used for the analysis of changes in shRNA pool composition between doxycycline-induced and non-induced samples over time.

#### shRNA Competition Assay

Kasumi-1 pSLIEW and SKNO-1 pSLIEW cells were transduced with pLKO5d.SFFV.miRNA30n with different expression marker for each target (dTOMATO representative of shCCND2 and shRE expression and RFP657 representing shNTC expression) (Schwarzer et al., 2017). Four days post-transduction, cells were analysed by flow cytometry (BD FACSCalibur). Each shCCND2 construct was mixed with shNTC in a mixture of 50% each. The percentage of shRNA-expressing cells was measured every 2-3 days by flow cytometry. *In vivo* approach was done using the same proportion of cells mixture. A dose of 2.5 x 10^5^ of cells mixture were intrahepatically injected into 1 to 4-day-old *Rag2*^*−/−*^*Il2rg*^*−/−*^129×Balb/c (RG) mouse. Tumour engraftment was assessed via bioluminescent imaging (IVIS Spectrum, Caliper with Living Image Software). Tumour was harvested and analysed with flow cytometry at endpoint of experiment.

#### *In Vivo* Palbociclib Treatment

Kasumi-1 pSLIEW cells were intrahepatically injected into 17 newborn (1-4 days old) immunodeficient *Rag2*^*−/−*^*Il2rg*^*−/−*^129×Balb/c (RG) mice at a cell dose of 2.5 x10^5^ cells/mouse as described previously (Martinez Soria et al., 2009). From 5 weeks old, cell engraftment was assessed weekly via bioluminescent imaging (IVIS Spectrum, Caliper with Living Image Software). Mice were randomised into two treatment groups, one given palbociclib 100 mg/kg once daily and the other water vehicle at 10 μl/g body weight, orally by gavage in an unblended fashion. Treatment was given in 3 blocks of 7 days separated by 7 day blocks with no treatment. This schedule was found to be well tolerated with little weight loss compared to controls.

Engraftment of secondary mouse AE9a transplants in C57BL/6 mice was monitored by flow cytometry. As soon as flow cytometry indicated 5% total GFP+ or 2% GFP+ KIT+ (as a % of live cells) in peripheral blood, secondary recipients were treated with control vehicle (CV) or palbociclib by oral gavage in the following treatment blocks: day 9 to day 18, 150 mg/kg/day; day 42 to day 46, 100mg/kg/day; day 49 to day 50, 100mg/kg/day. Secondary recipient mice were monitored closely for signs of leukemia (behavioral, body condition, weight loss, piloerection, hind limb paralysis). Since 150 mg/kg/day approached the Maximal Tolerated Dose in this model, we reduced the dose for the final two blocks to 100 mg/kg/day. In addition, automated cell counts (analyzed on a Sysmex XT 2000i analyzer) and flow cytometric analyses (using an LSRFortessa X20 analyzer) were performed on tail vein blood microsamples, collected using heparinized end-to-end Micro Pipettes (Vitrex). The reagents used in the flow cytometric analyses were: 1μg/ml anti-mouse CD117 (c-Kit) APC-eFluor 780 (ThermoFisher Scientific, eBioscience clone 2B8); 1 μg/ml anti-mouse CD45.2 PerCPCy5.5 (ThermoFisher Scientific, eBioscience clone 104); 0.68 μg/ml anti-mouse CD45.1 eFluor 450 (ThermoFisher Scientific, eBioscience cloneA20); 1 μg/ml Hoechst 33258, Pentahydrate (bis-Benzimide) (ThermoFisher Scientific) for live/dead staining.

#### CHIC Analysis of Long-Range Promoter Contacts

Kasumi-1 cells (5× 10^7^) were fixed in 37 ml of RPMI-1640 supplemented with 15% FBS and 2% formaldehyde for 10 minutes at room temperature. 6 ml of 1M glycine (0.125 M final concentration) was added to quench the reaction and cells were incubated at room temperature for 5 min, followed by 15 minutes on ice before pelleting the cells at 4°C and washing them in ice cold PBS. Each sample was flash frozen in liquid nitrogen, and stored at −80°C. Cells were lysed in a tight dounce homogeniser (ten cycles) with 3ml of cold lysis buffer (10 mM Tris-HCl pH 8, 10 mM NaCl, 0.2% Igepal CA- 630, one tablet protease inhibitor cocktail (Roche complete, EDTA-free, 11873580001)). Cells were left on ice for five minutes then homogenised another ten times. The lysed cells, in 3 ml lysis buffer, were added to 47ml of lysis buffer and incubated on ice for 30 minutes with occasional mixing. Chromatin was pelleted and resuspended in 1ml of 1.25x NEBuffer 2 and split into four. Each sample was then pelleted at 1000 rpm and resuspended in 358 μl of 1.25x NEBuffer 2. 11 μl 10% SDS was added and each tube was incubated at 37°C for 60 minutes, rotating at 950 rpm. Samples were mixed by pipetting up and down every 15 minutes. SDS was quenched with 75μl 10% Triton X-100 and incubated at 37°C for 60 minutes. HindIII digestion, biotinylation, ligation, crosslink reversal, promoter capture and library preparation was performed exactly as described previously (Mifsud et al., 2015).

### Quantification and Statistical Analysis

#### Statistical Comparison of Experimental Groups

If not indicated otherwise, all statistical comparisons were performed using two-sided Student’s t-test.

#### Statistical Analysis of SHRNA Representation

Read counts for each dataset were normalised with upper-quartile normalisation method implemented in edgeR (Robinson et al., 2010). For *in vitro* SKNO-1 colony formation assays dataset, differential representation of shRNAs between doxycycline-induced and non-induced samples was assessed using edgeR. In brief, data were fit to a generalized linear model (GLM) and a GLM likelihood ratio test was performed to determine whether the coefficient representing the contrast between the conditions of interest was equal to zero, which indicate no differential representation.

For the other three *in vitro* datasets, time course analyses were performed in order to find shRNAs that have different responses between doxycycline-induced and non-induced arms over time with linear modeling using voom/limma pipeline (Law et al., 2014, Ritchie et al., 2015). For each dataset, the analysis was performed by fitting a temporal trend to time points/replatings for each condition using a natural regression spline function from the splines package. To test for any differences in the spline fits between induced and non-induced conditions, we created a model matrix that includes an interaction term corresponding to differences in the curves between conditions and a contrast matrix that is equivalent to the null hypothesis stating that the trends are equivalent between conditions taking into account any difference in the magnitude of expression and the shapes of the splines. Log2 fold changes between induced and non-induced samples were then calculated by subtracting the log2-transformed counts per million (CPM) value of the induced sample from the CPM value of non-induced sample at each time point for each shRNA.

For the *in vivo* datasets, RUVr approach from RUVSeq (Risso et al., 2014) was used to estimate unwanted variation, and in our case was potentially attributed by data generated from two different sequencing platforms. We used RUVr to calculate factors of unwanted variation using residuals from a first pass GLM regression of the upper-quartile normalised counts on the covariate of interest i.e. doxycycline induction effect. To adjust for this technical bias, the estimated factors of unwanted variation as well as the covariate of interest were both included in the model for differential representation analysis which was performed using the negative binomial GLM approach implemented in edgeR (Robinson et al., 2010). P values were adjusted to control for the false discovery rate (FDR) using the Benjamini-Hochberg method (Benjamini and Hochberg, 1995).

#### Gene Test and Ranking

The modified robust rank aggregation (α-RRA) module implemented in MAGeCK (Li et al., 2014) was employed to identify essential genes, which are genes with many shRNAs ranked near the top of the shRNA list sorted by P-values from the shRNA differential representation analysis procedure described above. In brief, α-RRA looks for genes whose shRNA rankings are consistently higher than expected and computed the statistical significance of the skew in ranking by permutation.

#### Differential Gene Expression Analysis

For expression profiling with RNA-seq data, paired-end reads were mapped to the reference human genome hg19 using STAR2-pass allowing up to two mismatches (Dobin et al., 2013). Per gene raw read counts for each sample were obtained using HTseq and Gencode version 19 (Anders et al., 2015). Gene-level differential expression analysis was performed using DEseq2 (Love et al., 2014). P-values were adjusted to control for the false discovery rate (FDR) using the Benjamini-Hochberg method (Benjamini and Hochberg, 1995). Gene set enrichment analyses were performed using MSigDB (Subramanian et al., 2005).

#### Analysis of Published AML Microarray Datasets

The microarray dataset (GSE6891) was downloaded from the Gene Expression Omnibus (GEO) using the Bioconductor GEOquery package (Davis and Meltzer, 2007). Expression data were processed and normalised with Robust multi-array average (RMA (Gautier et al., 2004)) using the Bioconductor affy package (Irizarry et al., 2003). CCND2 gene expression was calculated from the mean average expression of probes targeting the gene. P-value calculated using two-sided Wilcoxon test indicates significant difference of CCND2 expression between the groups.

### Data and Software Availability

The accession number for the CHiC and JUND ChIP data reported in this paper is GEO: GSE117108. ChIP-seq, DHS-seq, RNA-seq and bead array data have been previously deposited under GSE29225 and GSE60121 (Ptasinska et al., 2012, Ptasinska et al., 2014).

## Supplemental Information

Document S1. Figures S1–S8

Table S1. RNAi Library shRNA Sequences, Related to Figure 1

Table S2. RNAi Screen *In Vitro* Next-Generation Sequencing Reads, Related to Figure 1

Table S3. RNAi Screen *In Vivo* Next-Generation Sequencing Reads, Related to Figure 1

Table S4. Patient Sample Number, Age, Gender, Chromosomal Abnormalities, Related to STAR Methods

Table S5. qPCR Primer Sequences, Related to STAR Methods

Document S2. Article plus Supplemental Information
