# Supplementary material for: The Oncogenic Transcription Factor RUNX1/ETO Corrupts Cell Cycle Regulation to Drive Leukemic Transformation
Source: Cancer Cell. 2018 Oct 8;34(4):626–642.e8. doi: 10.1016/j.ccell.2018.08.015 (PMC6179967; doi:10.1016/j.ccell.2018.08.015)
Supplement: Document S1. Figures S1–S8 [file mmc1.pdf]

Cancer Cell, Volume *34*

Supplemental Information

The Oncogenic Transcription Factor RUNX1/ETO Corrupts Cell Cycle Regulation to Drive Leukemic Transformation

Natalia Martinez-Soria, Lynsey McKenzie, Julia Draper, Anetta Ptasinska, Hasan Issa, Sandeep Potluri, Helen J. Blair, Anna Pickin, Asmida Isa, Paulynn Suyin Chin, Ricky Tirtakusuma, Daniel Coleman, Sirintra Nakjang, Salam Assi, Victoria Forster, Mojgan Reza, Ed Law, Philip Berry, Dorothee Mueller, Alex Elder, Simon N. Bomken, Deepali Pal, James M. Allan, Gareth J. Veal, Peter N. Cockerill, Christian Wichmann, Josef Vormoor, Georges Lacaud, Constanze Bonifer, and Olaf Heidenreich

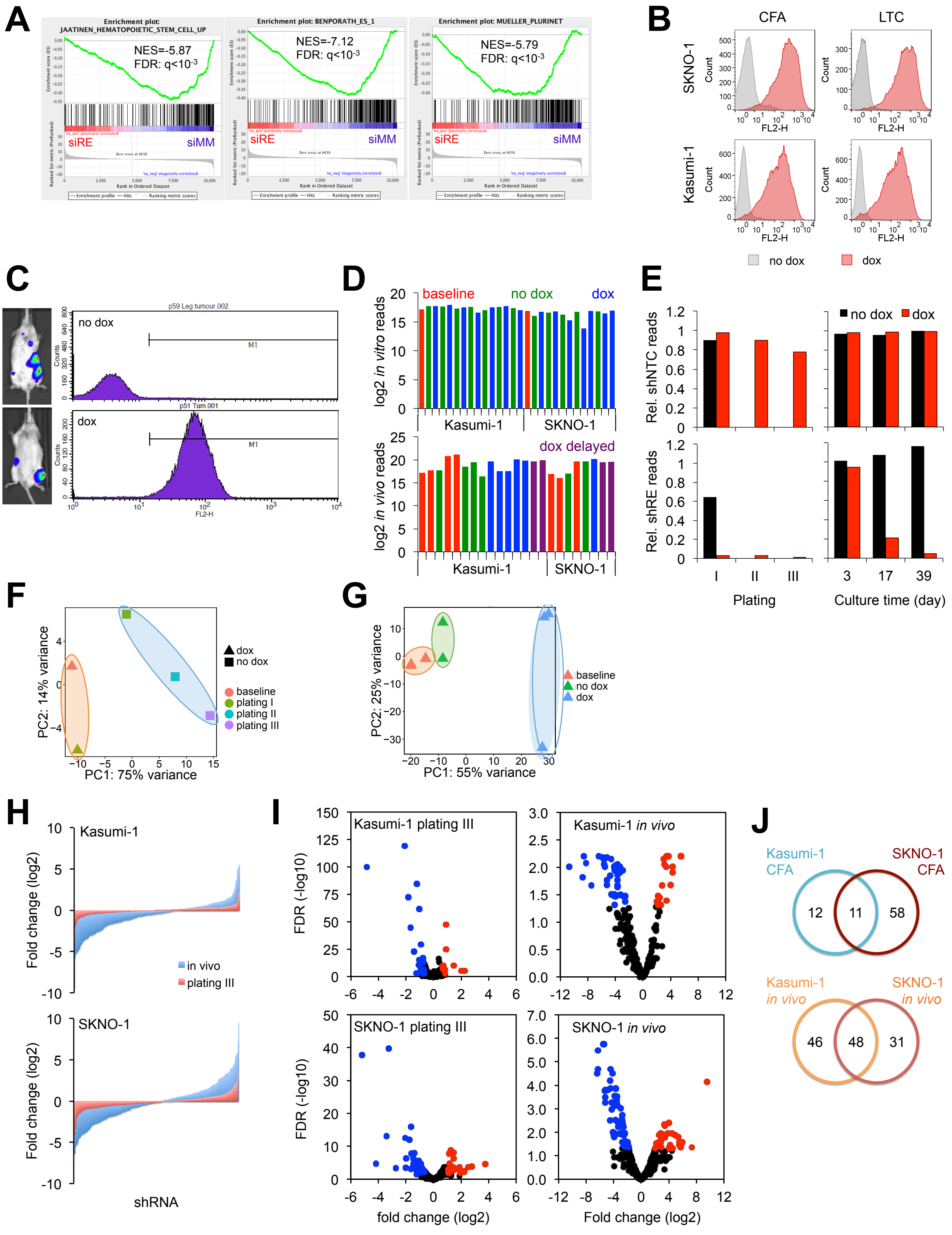

Figure S1 (related to Figure 1): A combined *in vitro*/*in vivo* RNAi screen identifies *CCND2* as crucial RUNX1/ETO transmitter.

(A) Gene set enrichment analysis (GSEA) with gene expression data obtained from RUNX1/ETO knockdown and mismatch siRNA-treated control cells showing enrichment of published gene sets associated with self-renewal (Ben-Porath et al., 2008; Jaatinen et al., 2006; Muller et al., 2008). siRE, siMM, treatment with RUNX1/ETO or mismatch siRNA, respectively. siMM, mismatch control siRNA; siRE, RUNX1/ETO siRNA (B) Histograms showing induction of shRNA-coupled RFP expression by doxycycline in SKNO-1 (top panels)

and Kasumi-1 cells (bottom panels) after replating (CFA, left column) or long-term suspension culture (LTC, right column). Grey peaks, no induction of shRNA expression; red peaks, induction of shRNA expression by doxycycline. (C) Histograms showing shRNA induction in Kasumi-1 *in vivo* as determined by FACS. No dox, no induction; dox, shRNA induction with doxycycline. Left, IVIS bioluminescence analysis of the corresponding NSG mice showing similar engraftment of Kasumi-1 cells, which have been transduced with shRNA library. M1, region of RFP positivity; p, mouse identifier. (D) Graphs showing overall NGS read count numbers for shRNA libraries in *in vitro* (top) and *in vivo* (bottom) arms of RNAi screen. Baseline, initial sample before induction with doxycycline; dox delayed, doxycycline induction 28 days after transplantation. (E) Graphs showing changes in non-targeting negative control shRNA (shNTC, top panels) and the positive control RUNX1/ETO shRNA (shRE, bottom panel) in SKNO-1 cells over three replatings (plating, left column) or a time course in long term suspension culture (right column). Black columns, no induction of shRNA expression; red columns, induction of shRNA expression by doxycycline. CFA was terminated for control cells after first plating. (F, G) Principal component analysis (PCA) of shRNA pools cells demonstrating separation of dox-induced shRNA pools (highlighted in blue) from non-induced and baseline pools (orange) during replating (F) and after xenotransplantation (G) of SKNO-1 cells. (H) Waterfall plots showing changes in shRNA construct levels in Kasumi-1 (top panel) and SKNO-1 cells after replating (orange) and engraftment in NSG mice (blue). (I) Volcano plots indicating shRNA log fold change in shRNA pool composition in Kasumi-1 and SKNO-1 cells. Blue and red points indicate significantly (FDR<0.1) depleted and enriched shRNAs with ≥1.5 and ≥2.0 change after third plating or engraftment *in vivo*. (J) Venn diagrams showing overlap of depleted shRNA constructs during replating (CFA, left) and engraftment (*in vivo*, right) between Kasumi-1 and SKNO-1 cells.

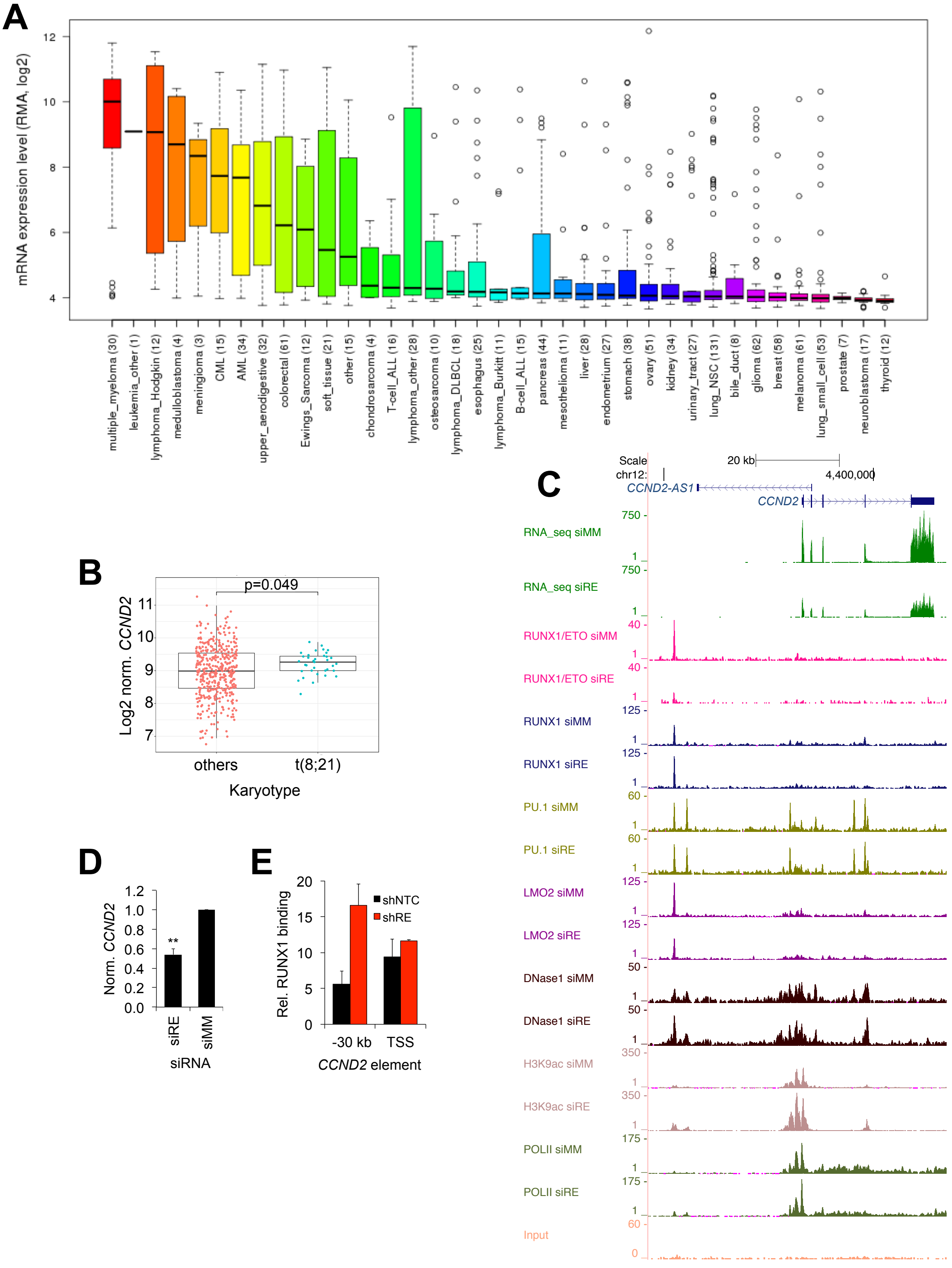

Figure S2 (related to Figure 2). RUNX1/ETO controls *CCND2* via a -30 kb regulatory element.

(A) Graph visualizing expression of *CCND2* across a range of malignancies. Data were obtained from the Broad-Novartis Cancer Cell Line Encyclopedia. Box and whisker plots show the distribution of mRNA expression for each subtype, ordered by the median CCND2 expression level (line), the interquartile range (box) and up to 1.5x the interquartile range (bars). Sample numbers (n) are indicated in parentheses. (B) Box and whiskers plot comparing *CCND2* expression between patients with t(8;21)-positive and negative AML. Line, median; horizontal box, interquartile range; whiskers, 1.5x interquartile range. p value was calculated by Mann–Whitney U test. Data were obtained from GEO gse6891. (C) Genome browser screen shot visualizing

RUNX1/ETO knockdown-induced changes in transcription factor binding (RUNX1/ETO, RUNX1, PU.1, LMO2), chromatin accessibility (DNase1) H3K9 acetylation and RNA pol II occupation at the *CCND2* locus as indicated by ChIP-seq, DHS-seq and RNA-seq (green). Scale denotes base pair position on chromosome. (D) Column graph showing diminished *CCND2* transcript levels upon RUNX1/ETO knockdown in Kasumi-1 as determined by qPCR. Mean ± SD; n=3-5. (E) Assessment of RUNX1 binding in control (shNTC) or RUNX1/ETO knockdown (shRE) Kasumi-1 cells at the -30 kb element of *CCND2* by manual ChIP. ***, p<0.001; **, p<0.01; *, p<0.05.

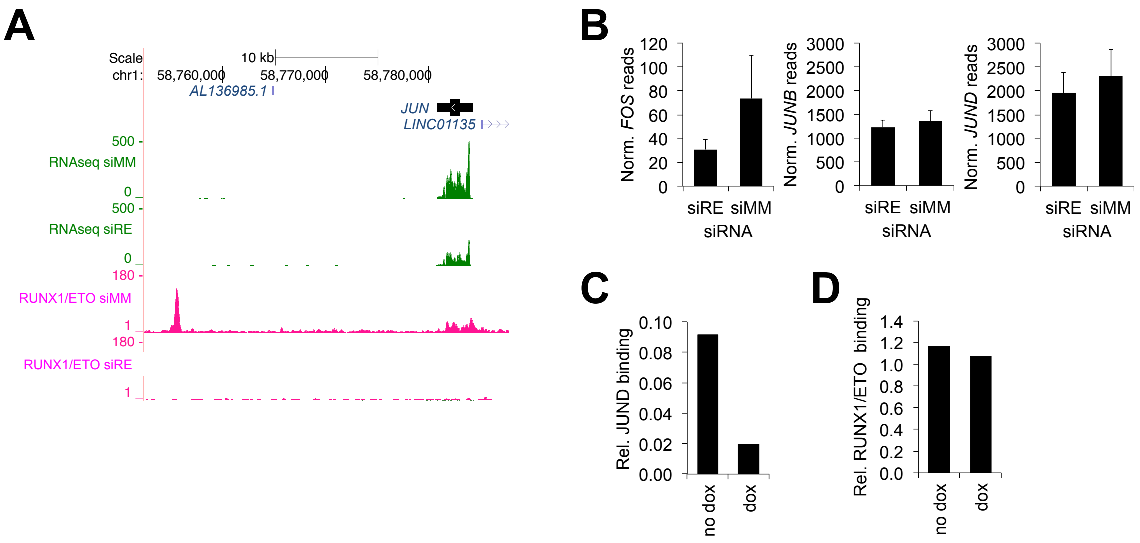

Figure S3 (related to Figure 3). RUNX1/ETO regulates *CCND2* expression by modulating AP1.

(A) Genome browser screen shot showing RUNX1/ETO binding of the *JUN* locus and *JUN* transcript level by ChIP-seq (pink) and RNA-seq (green). siMM, mismatch control siRNA, siRE, RUNX1/ETO siRNA. Scale denotes base pair position on chromosome (B) Graphs depicting changes in transcript levels of indicated *JUN* and *FOS* members upon RUNX1/ETO knockdown by RNA-seq. n=3. (C) ChIP-seq analysis depicting impact of dnFOS induction in Kasumi-1 by doxycycline on JUND binding to the *CCND2* promoter region. n=2. (D) Graph showing RUNX1/ETO binding to the -30 kb element with and without induction of dnFOS by doxycycline in Kasumi-1 by ChIP-seq.

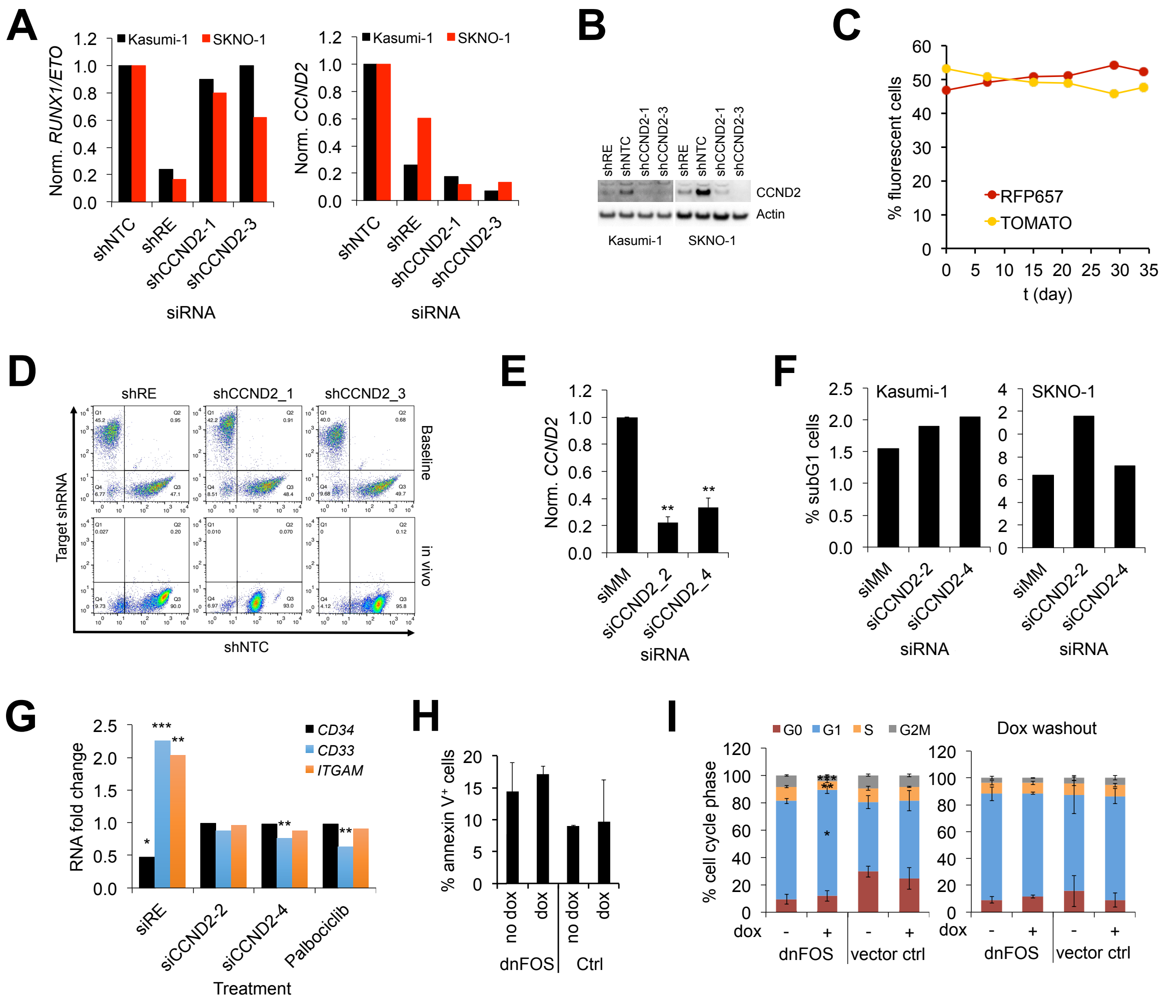

Figure S4 (related to Figure 4). RUNX1/ETO-expressing AML cells are addicted to CCND2.

(A) Graph depicting impact of shRNA transduction of shRE, shCCND2-1 and shCCND2-3 in Kasumi-1 and SKNO-1 cells on transcript levels of RUNX1/ETO (left panel) and CCND2 (right panel) as determined by qPCR. shNTC, nontargeting control shRNA; shRE, RUNX1/ETO shRNA; shCCND2-1 and -3, CCND2 shRNAs. (B) Western blot showing shRNA-mediated knockdown of CCND2 in lentivirally transduced Kasumi-1 and SKNO-1 cells in relation to control actin. (C) Graph showing percentages of fluorescent Kasumi-1 cells transcduced with empty vector controls during competitive culture as determined by FACS. RFP657, red fluorescent protein 657; Tomato, dTomato fluorescent protein. (D) Dot plots displaying depletion of shRE and shCCND2-transduced Kasumi-1 cells after competitive transplantation of immunodeficient RG mice as determined by FACS. shRE, shCCND2-1 and shCCND2-3 expression is detected by concomitant expression of dTomato (y-axis) and shNTC expression is detected by concomitant expression of RFP657 (x-axis). Baseline competition cell pool (intital starting 50:50 ratio) is compared to *in vivo* end point. (E) Column graph showing knockdown efficiency of two distinct CCND2 siRNAs in Kasumi-1 cells as determined by qPCR. Mean ± SD; n=3; **, p<0.01 vs siMM by two-sided Student's t-test. (F) % subG1 cells present after CCND2 knockdown, indicative of apoptosis, by siRNA in both Kasumi-1 and SKNO-1 as determined by PI staining of DNA content by FACS. siRE, RUNX1/ETO siRNA; siCCND2-2 and -4, distinct CCND2 siRNAs. (G) Graph showing impact of siRNA or palbociclib (30 nM, 72 hr) treatment of Kasumi-1 cells on transcript levels of indicated differentiation markers. Transcript levels were determined by RNA-seq 4 days after siRNA electroporation or addition of palbociclib. *, p_{adj}<0.05; **, p_{adj}<0.01; ***, p_{adj}<0.001 by two-sided Student's t-test. (H) Graph visualizing impact of dnFOS induction on apoptosis as measured by annexin V positivity. Ctrl, empty vector control. Mean ± SD; n=3. (I) Impact of dnFOS induction by doxycycline on the cell cycle distribution of Kasumi-1 cells as determined by PI staining of DNA content by FACS. Left panel, cell cycle distribution after incubation for 4 days with and without dox; right panel dox being removed after 4 days with cell cycle

distribution being determined at day 8. Mean ± SD; n=3; *, $p<0.05$; **, $p<0.01$; ***, $p<0.001$ by two-sided Student's t-test.

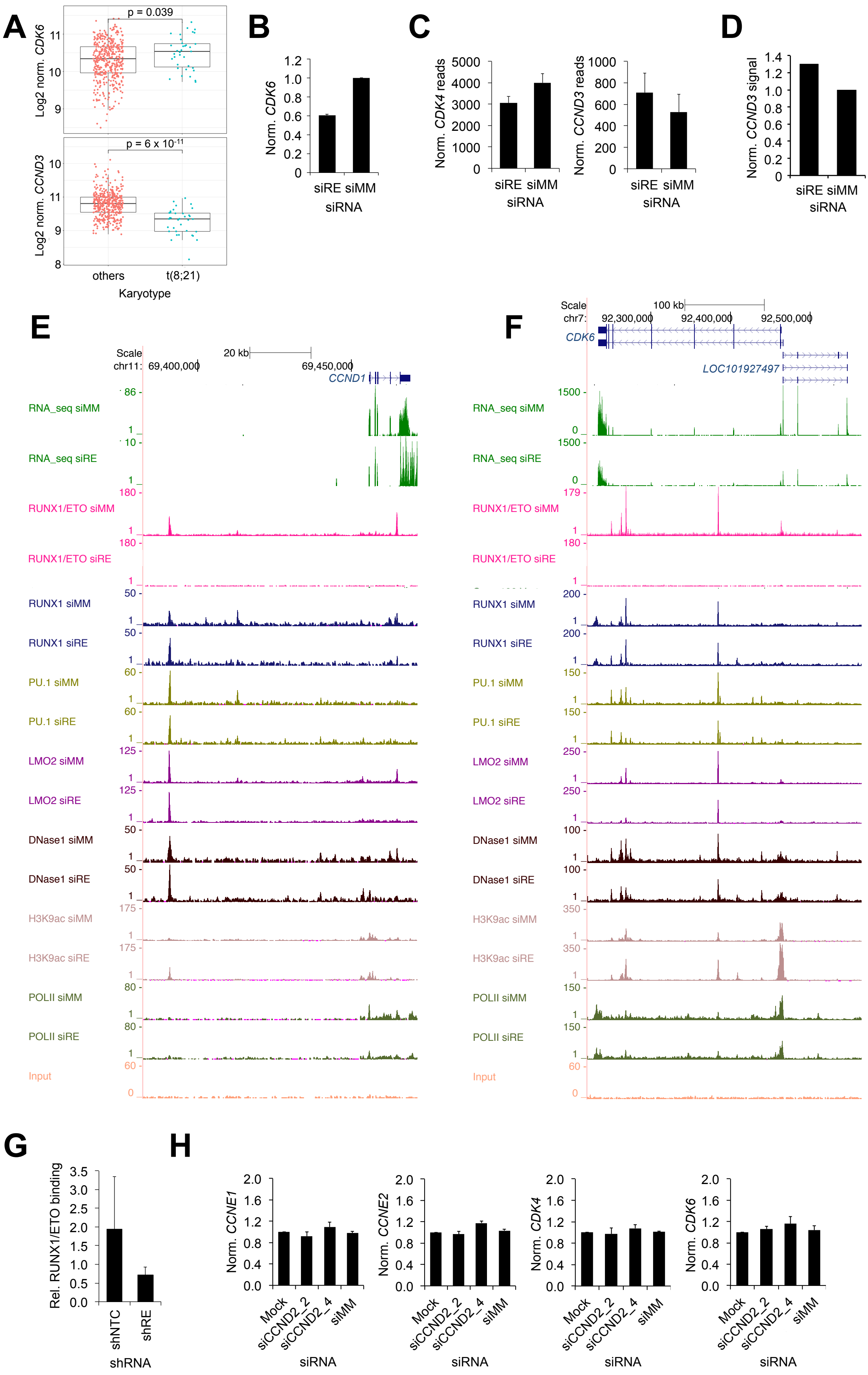
A
p = 0.039
Log2 norm. CDK6
p = 6 x 10-11
Log2 norm. CCND3
others
t(8;21)
Karyotype
B
Norm. CDK6
siRE siMM
siRNA
C
Norm. CDK4 reads
Norm. CCND3 reads
siRE siMM
siRNA
D
Norm. CCND3 signal
siRE siMM
siRNA
E
Scale
chr11:
20 kb
69,400,000
69,450,000
CCND1
RNA_seq siMM
RNA_seq siRE
RUNX1/ETO siMM
RUNX1/ETO siRE
RUNX1 siMM
RUNX1 siRE
PU.1 siMM
PU.1 siRE
LMO2 siMM
LMO2 siRE
DNase1 siMM
DNase1 siRE
H3K9ac siMM
H3K9ac siRE
POLII siMM
POLII siRE
Input
F
Scale
chr7:
100 kb
92,300,000
92,400,000
92,500,000
CDK6
LOC101927497
G
Rel. RUNX1/ETO binding
shNTC
shRE
shRNA
H
Norm. CCNE1
Norm. CCNE2
Norm. CDK4
Norm. CDK6
Mock
siCCND2_2
siCCND2_4
siMM
siRNA

Figure S5 (related to Figure 5). G1 cell cycle components are regulated by RUNX1/ETO, but do not compensate for CCND2 loss.

(A) Box and whiskers plots comparing *CDK4* and *CCND3* expression between patients with t(8;21)-positive and negative AML. Horizontal box lines indicate from the top third quartile, median and first quartile, whiskers indicate 1.5x interquartile range. p values were calculated by Mann–Whitney U test. Data were obtained from GEO gse6891. (B) Graph showing reduction of *CDK6* transcript levels upon RUNX1/ETO knockdown in Kasumi-1 cells as determined by real-time PCR. siRE, RUNX1/ETO siRNA; siMM, mismatch control siRNA. Mean ± SD; n=3. (C) Graph displaying *CDK4* and *CCND3* expression in Kasumi-1 with and without RUNX1/ETO knockdown as indicated by RNA-seq. Note the different scales for RNA-seq data of *CCND1*. Mean ± SD; n=3. (D) Graph displaying decreased CCND3 transcript levels in primary t(8;21) AML blasts upon RUNX1/ETO knockdown as analyzed by Illumina bead arrays with probe ILMN_1668721. n=1. (E, F) UCSC genome browser screen shot visualizing RUNX1/ETO knockdown-induced changes in transcription factor binding (RUNX1/ETO, RUNX1, PU.1, LMO2), chromatin accessibility (DNase1), H3K9 acetylation and RNA pol II occupation at the *CCND1* (E) and *CDK6* (F) loci in Kasumi-1 as indicated by ChIP-seq, RNA-seq (green) and DHS-seq (DNase1). (G) Chip validation of the loss of RUNX1/ETO binding to the *CDK6* locus upon RUNX1/ETO knockdown in Kasumi-1. shRE, RUNX1/ETO shRNA; shNTC, non-targeting control shRNA. Mean ± range; n=2. (H) Impact of CCND2 knockdown by two different siRNAs on mRNA levels of *CDK6*, *CDK4*, *CCNE1* and *CCNE2*. Kasumi-1 cells were sequentially electroporated every 2 days with the indicated siRNAs. Transcript levels were determined on day 8 by qPCR. siCCND2_2, _4, CCND2 siRNA. Mean ± SD; n=3.

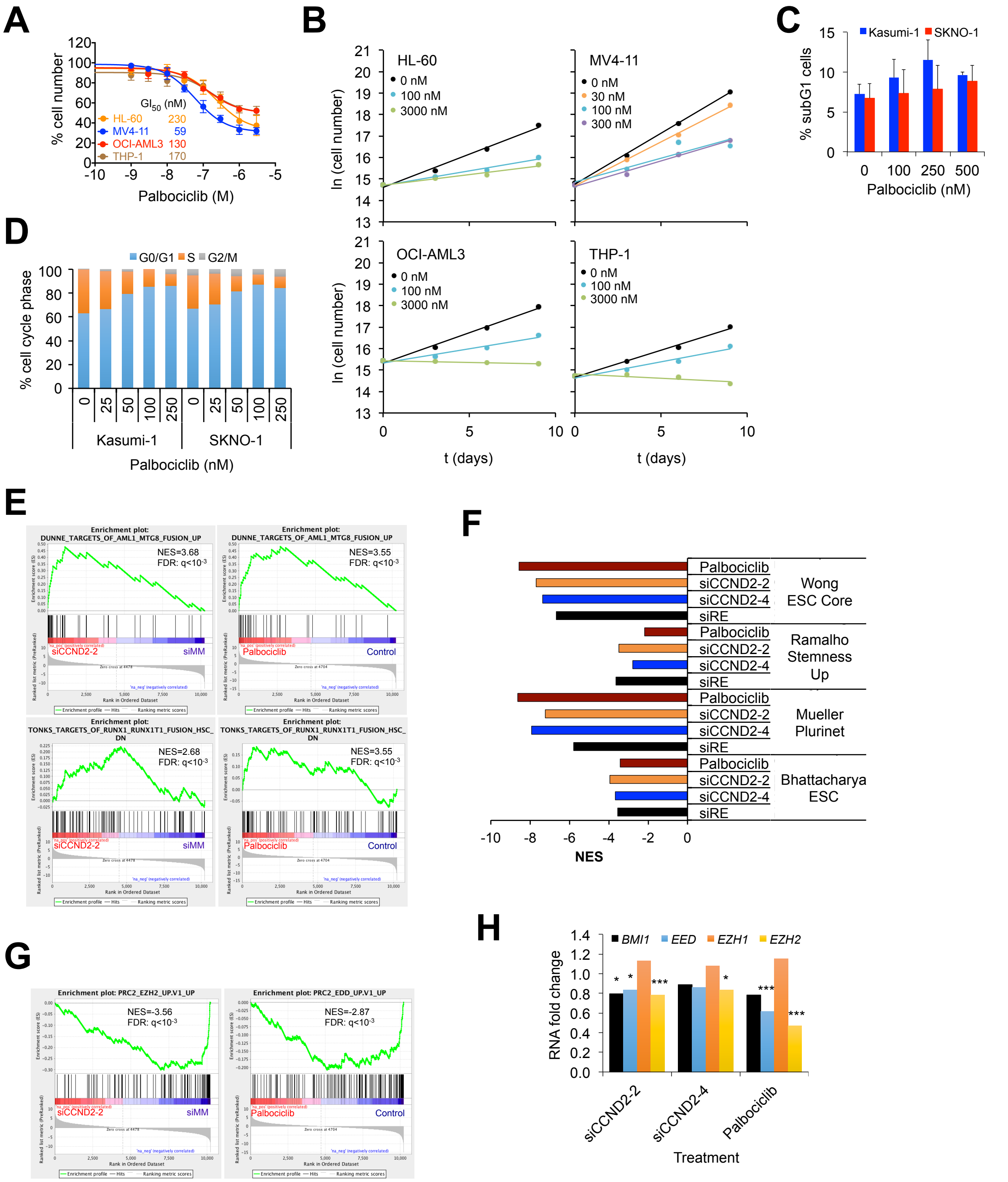

Figure S6 (related to Figure 6). The CDK4/6 inhibitor palbociclib inhibits growth of RUNX1/ETO-expressing leukemic cells.

(A) Growth curves of t(8;21)-negative AML cell lines with palbociclib for 72 hr. Mean ± SD; n=5. (B) Proliferation of t(8;21)-negative AML cell lines during long-term treatment with indicated concentrations of palbociclib. n=2. (C) Graph depicting changes in subG1 cell fractions as an indicator of apoptosis of Kasumi-1 and SKNO-1 cells after 72 h treatment with the indicated palbociclib doses as determined by PI staining and analysis by FACS. Mean ± SD; n=3. (D) Graph depicting cell cycle distribution of Kasumi-1 and SKNO-1 cells after 6 days of treatment with the indicated palbociclib doses as determined by PI staining and analysis by FACS. (E) GSEA with gene expression data obtained from CCND2 knockdown and palbociclib treatment of Kasumi-1 cells showing correlation between published RUNX1/ETO gene sets (Dunne et al., 2006; Tonks et al., 2007) and palbociclib and CCND2 knockdown signatures. (F) Bar diagram showing the normalized enrichment score for stemness and self-renewal signatures for palbociclib treatment, CCND2 and RUNX1/ETO knockdown

gene sets. NES, normalised enrichment score. (G) GSEA with gene expression data obtained from CCND2 knockdown and palbociclib treatment showing negative enrichment of PRC2 EZH2 and EDD oncogenic signatures. (H) Graph showing impact of CCND2 siRNAs or palbociclib treatment of Kasumi-1 cells on transcript levels of indicated polycomb genes (BMI1, EED, EZH1, EZH2). Transcript levels were determined by RNA-seq 4 days after siRNA electroporation or addition of palbociclib. *, p_{adj}<0.05; **, p_{adj}<0.01; ***, p_{adj}<0.001.

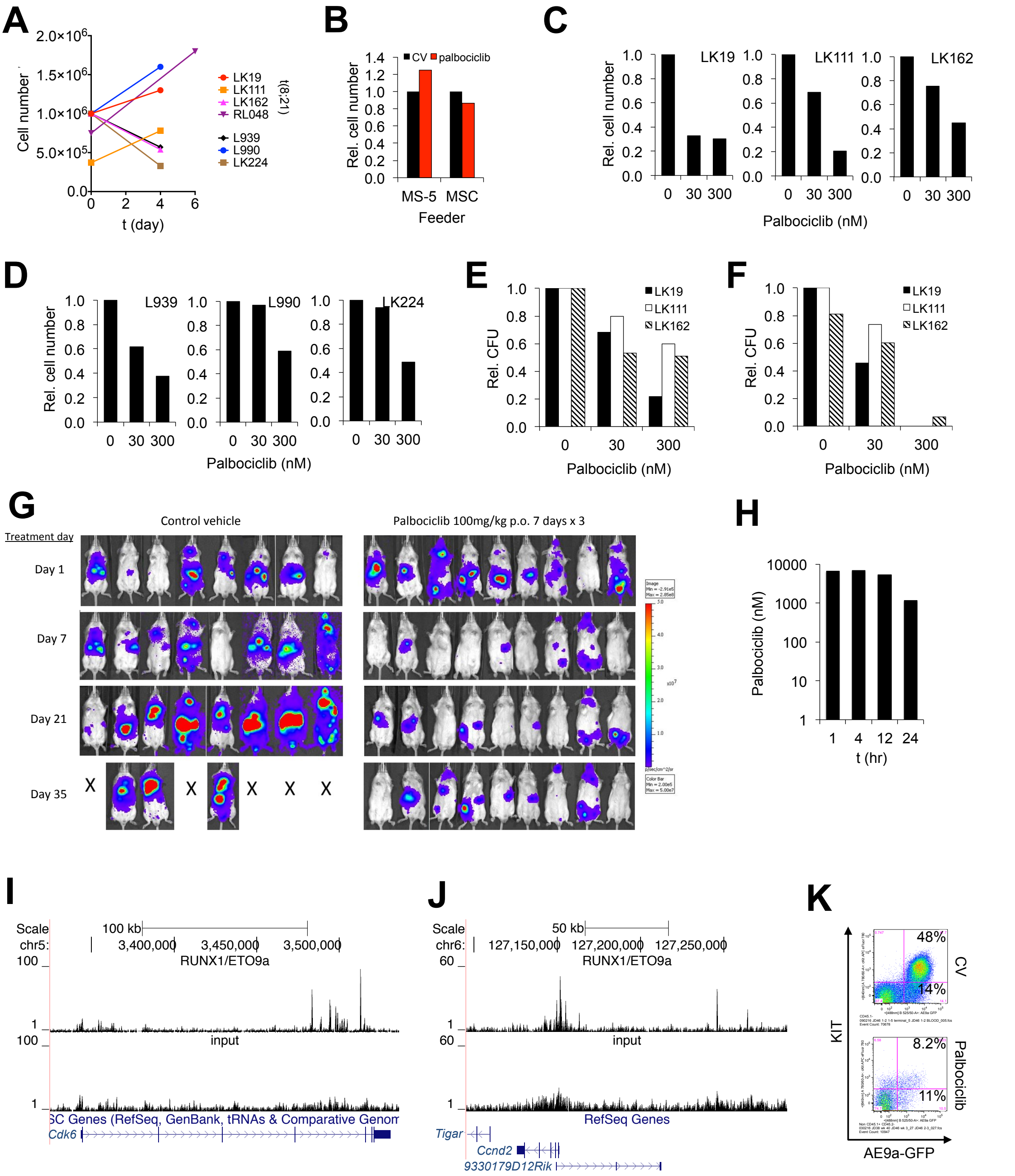

Figure S7 (related to Figure 7). CDK4/6 inhibition impairs proliferation of primary AML cells and increases median survival *in vivo*.

(A) Expansion of primary AML on MS5 (RL048) and MSC feeder layers (all other samples). Representative of n=2 (B) Impact of palbociclib (300nM) or CV on MSC and MS-5 feeder layer proliferation for 96 hr. CV, control vehicle. n=1 (C, D) Impact of palbociclib (300 nM) on proliferation of three t(8;21)-positive (C) and negative (D) AML patient samples, respectively. Patient cells were incubated on MSC feeder layers with and without (CV) 300 nM palbociclib. (E, F) Impact of palbociclib on colony formation of t(8;21) AML cells. Cells were first incubated on MSC layers followed by culture in semi-solid medium either without (E) or with (F) palbociclib. Colonies were counted after 12 days post-plating. (G) Images of RG mice transplanted with luciferase-expressing (luc$^+$) Kasumi-1 cells after the indicated days of treatment with control vehicle (CV) or palbociclib by IVIS bioluminescence imaging. Treatment blocks are indicated in Figure 7H and I. (H) Plasma levels of palbociclib. RG mice were treated ith a single dose of 100 mg/kg p.o. Plasma levels of palbociclib were determined by LC/MS analysis. (I, J) Genome browser screen shots visualizing binding of RUNX1/ETO9a to the murine *Cdk6* (I) and *Ccnd2* (J) loci. (K) FACS analysis of Kit+ RUNX1/ETO9a (AE9a) GFP+ cells in mice after CV or palbociclib treatment.

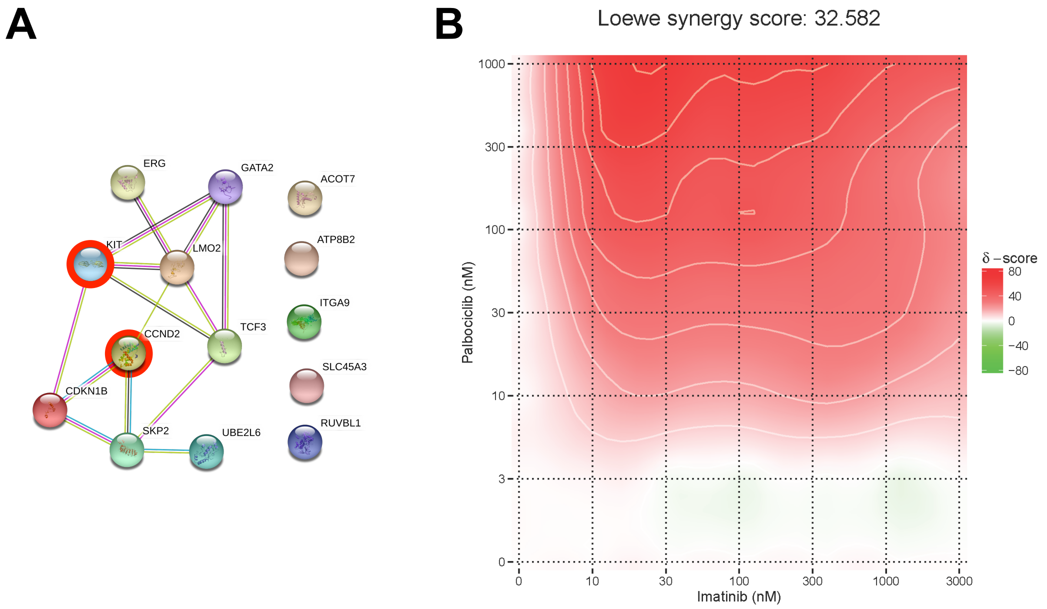

Figure S8 (related to Figure 8). CDK4/6 interference sensitizes AML cells towards inhibition of ribonucleotide reductase and mutated KIT.

(A) String-generated gene network showing interactions between genes indicated by CFA RNAi screens. Nodes represent genes indicated by at least two shRNAs in combined SKNO-1 and Kasumi-1 screens. (B) Surface blot showing synergy for palbociclib-imatinib combo in Kasumi-1 cells. Loewe additivity model is used. Loewe score indicates overall additivity of the two drugs across the matrix. Red, increased additivity; green, decreased additivity.
